# The Elastic Share of Inelastic Stress–Strain Paths of Woven Fabrics

**DOI:** 10.3390/ma13194243

**Published:** 2020-09-23

**Authors:** Mehran Motevalli, Jörg Uhlemann, Natalie Stranghöner, Daniel Balzani

**Affiliations:** 1 Chair of Continuum Mechanics, Ruhr-University Bochum, D-44801 Bochum, Germany; mehran.motevalli@rub.de; 2 Institute for Metal and Lightweight Structures, University of Duisburg-Essen, 45117 Essen, Germany; joerg.uhlemann@uni-due.de (J.U.); natalie.stranghoener@uni-due.de (N.S.)

**Keywords:** structural fabric, stress–strain behavior, viscoelastic material response, structural analysis, elastic modeling

## Abstract

Manifold variations of the mechanical behavior of structural woven fabrics appear in the first load cycles. Nevertheless, invariable states, i.e., mechanically saturated states, can be approached by multiple monotonous load cycle biaxial tests. In a state acceptably close to the ideal saturated state, the stress–strain paths reveal the elastic share of the initially inelastic stress–strain paths of woven fabrics. In this paper, the mechanical saturation behavior of two types of PTFE-coated woven glass fiber fabrics is examined and compared to the recently reported saturation behavior of a PVC-coated polyester fabric. With the help of the saturation test data, an extrapolation function is developed that facilitates an estimation of late cycle stiffness behavior based on measured early cycle behavior. Furthermore, the considerable impact of late cycle properties on structural analyses is shown exemplarily in the numerical simulation of a prestressed fabric structure by comparing results achieved from late and early load cycle stiffness parameters.

## 1. Introduction

Prestressed membrane structures increasingly deployed as roofs or facades are frequently made from woven coated fabrics. The load bearing fabrics in turn are usually made from polyester (PES) or glass fibers. In the framework of architectural applications, they are coated with materials like plasticized polyvinylchloride (PVC) or polytetrafluoroethylene (PTFE) to make them—among other properties—waterproof and resistant to environmental impacts. Under mono- or biaxial membrane stress states, these composites show a distinct nonlinear and visco-elastoplastic response. This behavior partly originates from the undulated yarn geometry being pulled straight under tension and partly from the viscoelastic and nonlinear material response of the utilized plastics of yarns and/or coatings. Inelastic effects are very pronounced in the first load cycles but reduce step-wise. However, they lead to manifold variations of the mechanical behavior: changes of the nonlinear form of both loading and unloading stress–strain paths, changes in the amount of irreversible strain after unloading, strain-rate dependency, etc.

Different approaches were made to model material nonlinearity and visco-elastoplastic effects in both principal directions of the fabric, i.e., warp and fill, and additionally to integrate shear stiffness, e.g., [1,2,3,4,5,6]. However, they are usually costly to handle and focus on specific aspects and time scales of the material response of coated fabrics. None are well-established in design practice by now and available in commercial software and thus it is common to perform simplified linear elastic structural analyses of fabric structures. Sets of fictitious anisotropic elastic constants are used for the linear elastic modeling [7]. In the framework of the elastic concept, inelastic effects have to be taken into account separately where required. A recent advancement in nonlinear elastic modeling was presented by the authors [8] achieving good fits with four easy-to-handle stiffness parameters.

This contribution aims to support the further development of elastic and visco-elastoplastic material models for orthotropic fabrics by extraction and demonstration of the late cycle behavior component. A precise separation facilitates the quantification of the single effects. Invariable states, i.e., mechanically saturated states can be approached by (biaxial) tests with multiple monotonous load cycles [9]. In a state acceptably close to the ideal saturated state of the stress–strain paths—which is only achieved after infinite load cycles—the elastic share of the initially inelastic stress–strain paths of woven fabrics is revealed.

In this paper, the mechanical saturation behavior and elastic share of the stress–strain response of two types of PTFE-coated woven glass fiber fabrics are examined and compared to the recently reported saturation behavior of a PVC-coated polyester fabric [9]. The saturation behavior is found to be similar to the PES-PVC fabric behavior. The irreversible strain increments settle fast and stay on a low constant level but the overall slope and nonlinear form of the stress–strain paths require more than 1000 load cycles to be saturated [9].

Moreover, an extrapolation function is developed that facilitates an estimation of late cycle stiffness behavior based on measured early cycle behavior. It is illustrated that the method can be applied with acceptable accuracy for an estimation of the saturation of different fabrics made of the same material components, i.e., for instance the behavior of a type III glass-PTFE fabric can also be estimated with test data of a type IV glass-PTFE fabric. Furthermore, it is exemplarily shown by a numerical simulation of a prestressed fabric structure that stiffness properties gained from different load cycles can lead to considerably different structural analysis results. This illustrates the importance of correct handling of stiffness properties.

## 2. Material Properties and Experimental Methods

Saturation tests, i.e., mono- or biaxial tensile tests with numerous identical load cycles, are suitable to extract the elastic share in the stress–strain paths of fabrics [9,10,11]. In the present contribution, biaxial saturation tests are performed with 1000 load cycles. Two different types of PTFE-coated glass fiber fabrics are investigated, representing frequently used types III and IV [7]. Basic properties of the tested materials according to producer declarations are provided in Table 1. The thin PTFE coating is applied by passing the bare glass fiber fabric through liquid PTFE dispersion and sintering the composite afterwards. Only coated fabric is investigated here as uncoated glass fiber fabric is not fit for architectural service. For later comparisons, a PES-PVC fabric investigated in [9] is also taken into account. It is a type III PES-PVC with tensile strength $f_{t}$ of 130 kN/m and 114 kN/m in the warp and fill directions, respectively.

The cyclic loading saturation tests are performed for five different stress ratios warp:fill, namely: 1:1, 2:1, 1:2, 1:0 and 0:1. For each stress ratio, a separate test specimen is used. The maximum stress in all load cycles is chosen as 30 kN/m for the fabric type III and 40 kN/m for the fabric type IV. These numbers each reflect one quarter of the warp tensile strength and cover the service stress typically applicable in architectural structures. The minimum stress is chosen to be close to zero. All tests are performed on the two biaxial test rigs available in the Essen Laboratory for Lightweight Structures, part of the Institute for Metal and Lightweight Structures at the University of Duisburg-Essen. Both rigs comply with EN 17117-1 [12], Annex A.4. The plane cross-shaped test specimens are characterized by long arms. Close to the 200 mm${}^{2}$ central measurement field they are slitted parallel to the arm length. The slits provide a homogeneous strain field. Orthogonal forces are applied to the arms in warp and fill direction via hydraulic actors. In-plane deformation and strain are measured contactless with a high precision one-camera optical system tracing four markers placed at the corners of a 100 mm${}^{2}$ square within the measurement area [13]. The measurement uncertainty is in the magnitude of 10 μm. For the present investigation, this is a minimum factor of 20 lower than the measured variables and is thus neglected in the interpretation of results. The load rate is 15 kN/m·min. Thus, the duration of each test is more than 66 h and 88 h for the both types of fabric, respectively.

**Figure 1:**
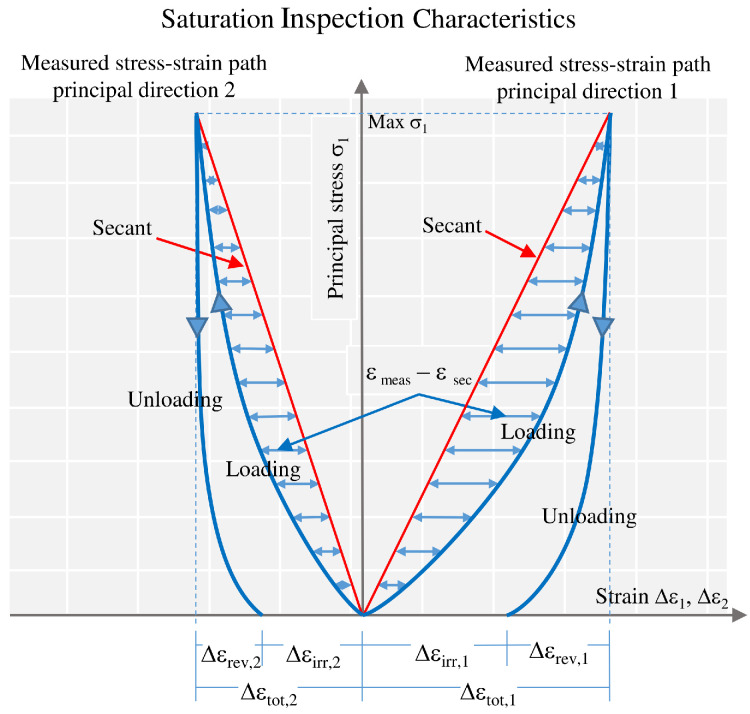
Saturation inspection characteristics, illustrated at normalized stress–strain paths in principal directions 1 and 2.

The saturation analysis of both fabrics is performed using the method proposed in [9]. Three inspection characteristics are defined which are computed for each load cycle and whose progress over the load cycles can be traced and quantitatively assessed. They are: (a) irreversible strain increment $\Delta \varepsilon_{\mathrm{irr}}$, (b) total strain increment $\Delta \varepsilon_{\mathrm{tot}}$ and (c) intensity of nonlinearity η, see Figure 1. Regarding the intensity of nonlinearity, η is calculated with a usual least squares approach with each summand being a difference between measured strain and calculated strain along the secant at predefined equidistant stress levels. The inspection characteristics are determined for both principal directions 1 and 2, with principal direction 1 being the direction subjected to the major stress of a given stress ratio, except in the even biaxial stress ratio 1:1 where principal direction 1 equals warp direction by definition. Ideal elasticity is present when (a) the irreversible strain increment converged to zero and (b) the total strain increment and (c) the intensity of nonlinearity converged against constant values. Taking advantage of these inspection characteristics, it can be evaluated how close the fabric behavior comes to the elastic, fully saturated state. For the PVC-coated polyester fabric investigated in [9], the saturated state was not reached after the tested number of 1000 load cycles. To achieve it, the curves of the inspection characteristics plotted against the load cycle number were fitted by a polynomial asymptotic function and extrapolated with the help of the found function. The saturated state was defined to be achieved when the irreversible strain increment becomes smaller than 1% of the total strain increment in the same load cycle and when additionally, the total strain increment and the intensity of nonlinearity approximate “their” asymptotes of the fitted progression functions with an acceptable error of less than 3%.

Additionally, in this contribution, material stiffness parameters are computed for selected load cycles to examine how the saturation progress manifests itself here. Two different elastic constitutive models will be considered. First, the widely used orthotropic linear elastic model, defined by four parameters: two orthogonal elastic moduli E${}_{x}$ and E${}_{y}$ and two Poisson’s ratios $\nu_{xy}$ and $\nu_{yx}$ [7]. Second, a new nonlinear polyconvex orthotropic material model is used as proposed in [8,14]. According to [8,15], the model can be described by a maximum of four material parameters related to a specific type of material which have to be fitted only once per material type. Another four model parameters are predetermined by taking into account stress–strain paths of all membrane types, see [15]. Hence, the design engineer does not have to handle more parameters than in the linear elastic model. In general, the nonlinear model provides significantly better reproduction of the measured material behavior than the linear one.

Due to considerable geometric nonlinearity of tensile structures and because all four parameters in both constitutive models change during the saturation process, the impact of variations on structural analyses cannot be assessed intuitively. Therefore, their impact is examined in a structural analysis of an exemplar prestressed fabric structure using Abaqus [16]. Therein, a pre-stretched large-scale roof structure subjected to realistic boundary conditions is modeled, see [8]. To illustrate the impact, different responses of the adjusted models to the selected states during the saturation process will be compared.

## 3. Saturated Elastic State of Glass-PTFE

**Figure 2:**
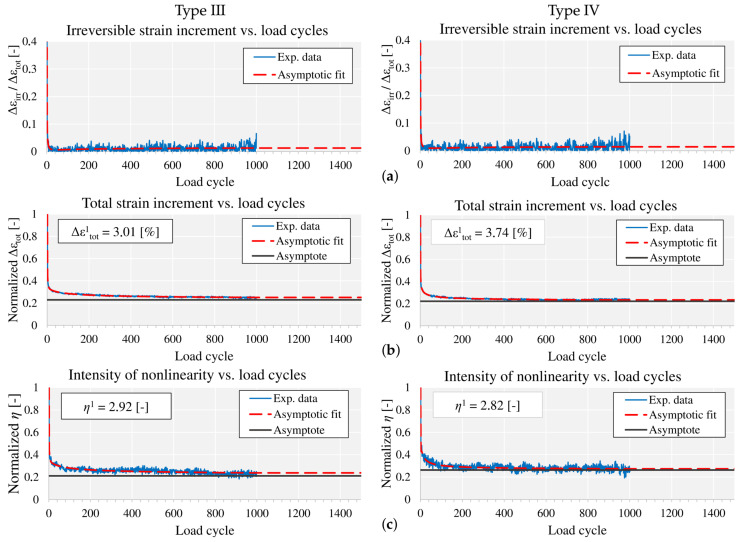
Representative curves of the inspection characteristics for the investigated glass-polytetrafluoroethylene (PTFE) fabric types III and IV: (**a**) irreversible strain increment; (**b**) total strain increment; (**c**) intensity of nonlinearity.

Figure 2 depicts exemplary curve progressions of all three inspection characteristics of the glass-PTFE fabric type III and IV, stress ratio 1:0, principal direction 1. They are shown here as representative for the saturation behavior of the tested glass-PTFE fabrics. A compilation of all diagrams is provided as supplementary data to this paper (Figures S1–S10). The saturation behavior of both investigated glass-PTFE fabrics is similar. A fast decrease of most inspection characteristics is observed in early load cycles, turning into a constant behavior (irreversible strain increment) or a very slow approximation of the ideal saturated state represented by the asymptote (total strain and intensity of nonlinearity). In single situations, the curve progression rather equals a sideways movement. This happens when the start value at load cycle one is already very small. To be able to recognize this, start values are given in the diagrams for total strain increment and intensity of nonlinearity. The irreversible strain increment is given related to the total strain increment. It was reported in [9] for a PES-PVC fabric that after the large decrease down to lower than 1% it turned into a constant magnitude, scattering around 1%. For the glass-PTFE fabrics investigated here, qualitatively the same is observed. Taking into account all investigated stress ratios, it can be said that after 10 ± 4 load cycles the value drops a first time to under 1% and scatters around a fairly constant mean afterwards. Compared to the PES-PVC fabric, bigger scatter and higher mean values are found. In most situations, the mean value is in a range of up to 2.5%. In single cases like for instance stress ratio 1:1, principal direction 1, it is found to be up to 5%. This is not because the irreversible strain is big. The percentage is rather high because total strain as the reference value is very small. However, the fact that the numbers do not become zero shows that the stepwise increase of irreversible strain never stops completely. The increments are small, even under the applied extreme load conditions with numerous repetitions of full permissible stress cycles, a situation which is not near to real structures exposed to natural loads.

Total strain increment as well as intensity of nonlinearity regularly strongly decrease to a magnitude of roughly 20% of the initial value in load cycle 1. The first property reveals the strongly increasing stiffness and reflects the high stiffness of the glass fibers in the saturated state after pulling out most of the yarn crimp. The second one indicates the approach to the typical linear stress–strain behavior of glass. Compared to the behavior of the polyester fabric reported in [9], considerably higher intensities of nonlinearity are observed for the glass fiber fabrics, particularly in the stress–strain paths of fill direction.

Overall, both investigated fabrics show a saturating mechanical behavior which converts them into an elastic state. Taking advantage of the saturation criteria defined in [9], saturated state load cycles are found for the glass-PTFE fabrics as provided in Table 2. Similar to the aforementioned polyester fabric, it is observed that the irreversible strain increment is quickly becoming constant while the total strain increment and the intensity of nonlinearity often require many thousands of load cycles to appropriately approach the saturated state—in a single case even millions.

In laboratory practice, it is not reasonable to perform biaxial tests with many thousands of load cycles to estimate the elastic share of a fabric’s response. Thus, an extrapolation method enabling to predict the elastic share from measurements of only a few load cycles is promising.

## 4. Elastic Material Models for Textile Membranes and Parameter Adjustment

The material behavior of woven coated fabrics becomes almost elastic after application of a large number of cyclic loadings [9,17]. As described in the previous sections, the irreversible portion of strain may decrease from one cycle to another and thus, the material response can be fairly simulated using elastic material formulations. Accordingly, the focus is given to the description of two elastic material models which are considered in the remaining analysis. First, the simple linear elastic material formulation is explained which is vastly used in industrial applications. Later, the recently proposed nonlinear orthotropic material model is described and its advantages are discussed. Subsequently, the material parameters of both formulations will be adjusted to different states of the tested fabrics. As a result, the change in the value of adjusted parameters may illustrate, to a good extent, the development of material response during the course of 1000 load cycles.

### 4.1. Linear Elastic Material Model

For a descriptive mathematical model of tensile fabrics using linear elastic formulation, the physical body is idealized as a two dimensional membrane with plane stress conditions and orthotropic material response [18,19]. In general, 5 elastic constants appear in the formulation which are identified from the stress–strain paths of the considered experiments. To this end, a typical least-square objective function may be introduced to minimize the difference between the experimental and computed engineering strains along the yarn directions; i.e., $\varepsilon_{w}$, $\varepsilon_{f}$ which are obtained from

(1) $$\varepsilon_{w}=\frac{n_{w}}{E_{w}t}-\nu_{\mathrm{fw}}\frac{n_{f}}{E_{f}t},\;\;\varepsilon_{f}=\frac{n_{f}}{E_{f}t}-\nu_{\mathrm{wf}}\frac{n_{w}}{E_{w}t}.$$

Another alternative of the objective function may be an error function which reduces the difference between the experimentally measured and numerically computed nominal stress values; i.e., $n_{w}$, $n_{f}$, which are formulated as

(2) $$n_{w}=\frac{E_{w}t}{1-\nu_{\mathrm{wf}}\nu_{\mathrm{fw}}}\left(\varepsilon_{w}+\nu_{\mathrm{fw}}\varepsilon_{f}\right),\;\;n_{f}=\frac{E_{f}t}{1-\nu_{\mathrm{fw}}\nu_{\mathrm{wf}}}\left(\varepsilon_{f}+\nu_{\mathrm{wf}}\varepsilon_{w}\right).$$

In these equations, $E_{w}$ and $E_{f}$ are the Young’s modulus in the warp and the fill directions. The Poisson’s ratios are denoted as $\nu_{\mathrm{wf}}$ and $\nu_{\mathrm{fw}}$. The thickness *t* is set to 1 mm and thus, the nominal stresses are the resultant ones with the unit of force per length kN/m. The same applies to the unit of elastic moduli. Since in the experiments no shear test was carried out, the in-plane shear moduli may not be identified here and later in the structural simulations it will be set to a usual magnitude of $G_{\mathrm{fw}}=60$ kN/m, see [20]. Taking into account the material symmetry constraint, the relation in Equation (3) holds, and hence the total number of material parameters to be identified for the linear elastic model is reduced to 3. That is, the second Poisson’s ratio is computed from Equation (3) and not directly considered in the optimization.

(3) $$\frac{\nu_{\mathrm{wf}}}{E_{w}t}=\frac{\nu_{\mathrm{fw}}}{E_{f}t}.$$

In order to preserve the positive definiteness of material tangent of the linear elastic formulation, the product of the Poisson’s ratios is bounded with $\nu_{\mathrm{fw}}\cdot \nu_{\mathrm{wf}}<1$. In case of violation of this condition, the linear elastic model can not be directly used in numerical simulations. In addition, this restriction leads to a less precise representation of the observed lateral contractions during uniaxial tensile tests in either directions.

In the current work, unlike the traditional method of parameter adjustment of the fabric membranes, see [18,19], the Poisson’s ratios are not identified only based on the stress–strain relation of biaxial tensile tests. Therefore, by inclusion of the lateral contraction data of the uniaxial tension tests in the objective function, the model is trained and may allow for more crosswise interactions, cf. [8].

### 4.2. Orthotropic Nonlinear Polyconvex Material Model

In the following, the nonlinear orthotropic material model is briefly described. The model was originally proposed for the geometrically nonlinear simulation of glass-PTFE fabrics, see [14]; nonetheless, the capability of the model was also demonstrated in the representation of material response of PES-PVC fabrics in [15]. The terms included in the model are all polyconvex, thereby, a numerically robust and materially stable formulation is guaranteed, cf. [14,21,22]. The orthotropic nonlinear model was characterized and investigated in [14]; wherein, the advantages including increased numerical robustness and match with experimental data of inclusion of the polyconvex orthotropic term was demonstrated and compared to the other competitive formulations, see [23,24]. Comparing the model to the linear elastic formulation in [8], the accuracy of the proposed model in simulation of the newly proposed large-scale inhomogeneous test setting, the pressure chamber test, using glass-PTFE material was showcased. In [15], taking into account three types of PES-PVCs, the possible meaningful correlation between the material parameters of the nonlinear model and the tensile strength was established.

In the continuum mechanical description, taking into account the constituents of woven coated fabrics, either glass-PTFE or PES-PVC, the heterogeneous material is idealized as a continuum membrane with an isotropic matrix (coating) which is reinforced in two perpendicular in-plane directions, i.e., warp- and fill directions. Therefore, by postulating the existence of a strain energy function ψ, the proposed hyperelastic model is formed as an additive combination of different energy terms. It is assumed that each separate nonlinear term $\psi_{i}\left(C,\gamma \right)$ represents a specific behavior where the intensity of the intended response is simply controlled with only one linear coefficient $\alpha_{i}$; i.e., $\alpha_{i}\psi_{i}\left(C,\gamma \right)$. This is the salient feature of the proposed nonlinear model, as at the end, only 4 material parameters $\alpha_{i}$ remain which are to be adjusted for an individual material, i.e., $\psi \left(\alpha ,C,\gamma \right)=\sum_{i=1}^{4}\alpha_{i}\psi_{i}\left(C,\gamma \right)$.

Each included nonlinear deformation mode $\psi_{i}\left(C,\gamma \right)$ is a function of the right Cauchy–Green deformation tensor $C=F^{T}F$, with F being the deformation gradient, and the nonlinear model parameters γ. The γ are internal parameters controlling the nonlinearity of each deformation mode. They are identified and fixed for each group of fabrics; i.e., PES-PVC and glass-PTFE, following [8] which is based on the proposed method in [25]. Thus, the final form of the nonlinear model becomes linear in the material parameters $\alpha_{i}$ while staying nonlinear in the deformations and is obtained in the following form

(4) $$\psi \left(\alpha ,C\right)=\alpha^{\mathrm{iso}}\psi^{\mathrm{iso}}\left(C\right)+\alpha_{w}^{\mathrm{ti}}\psi_{w}^{\mathrm{ti}}\left(C\right)+\alpha_{f}^{\mathrm{ti}}\psi_{f}^{\mathrm{ti}}\left(C\right)+\alpha^{\mathrm{int}}\psi^{\mathrm{int}}\left(C\right)+\epsilon \psi^{\mathrm{vol}}.$$

In the above, the isotropic term $\psi^{\mathrm{iso}}$ represents the isotropic response mainly caused by the coating (matrix) material. The two transversely isotropic terms act as tensile reinforcement in the warp- and fill directions which are denoted as $\psi_{w}^{\mathrm{ti}}$ and $\psi_{f}^{\mathrm{ti}}$. The orthotropic or interaction term mainly controls the crosswise inter-yarns interactions and is denoted with $\psi^{\mathrm{int}}$. In addition, one penalty/volumetric term is included which assures a nearly incompressible material response. Hence, the coefficient is chosen large enough to fulfill this purpose $\epsilon =1\times 10^{6}\;\mathrm{kN}/m$. Although, the terms in the energy density are included to represent the intended response, the impact of different terms on each other is not completely avoidable.

In the formulation of the implemented polyconvex orthotropic term, unlike the typical multiplicative non-polyconvex ones [23,26], the notion of anisotropic metric tensor G is utilized as the so-called structural tensor. In general, the full component metric tensors are able to describe generic classes of anisotropy which at the same time may preserve the polyconvexity condition of the energy density functions [22]. The anisotropic metric tensors G are, therefore, a more generalized form of the classical structural tensor which are defined as $M_{i}={\hat{a}}_{i}⊗{\hat{a}}_{i}$, cf. [27]. In the classical form, also utilized in the formulation of the transversely isotropic terms, here $\psi_{i}^{\mathrm{ti}}$, ${\hat{a}}_{i}$ is a unit vector pointing in the yarn direction, where the properties $|{\hat{a}}_{i}|=1$, $\mathrm{tr}\left[M_{i}\right]=1$ and i=w/f hold. However, the metric tensor is determined as $G:=HH^{T}$ with tensor H being a linear tangent map of a fictitious Cartesian basis ${\hat{e}}_{i}$ into the material principal directions; i.e., $H:{\hat{e}}_{i}\to {\bar{a}}_{i}$ and ${\bar{a}}_{i}=H{\hat{e}}_{i}$ where $i\in \left\{1,2,3\right\}$. For further details on the characteristics of anisotropic metric tensors, we refer to [14,22,28]. In the simplified case where the global Cartesian basis and the material basis coincides, the orthotropic metric tensor turns into the simple diagonal matrix $G=\mathrm{diag}\left[a^{2},b^{2},c^{2}\right]$ with only 3 non-zero components a,b,c which are the postulated length of material basis in the warp, fill and thickness directions, respectively. They can scale a material property in the specific material direction and at the same time, allow for direct transverse interactions. In the case of textile membranes where only two yarn families are presented, the thickness direction component may be set to zero c=0. For further simplification purpose, the component in $x_{1}$-direction is set to one, i.e., a=1, and only the model parameter *b* is to be identified for a specific material which will be termed as $\gamma_{4}$ in the model. Thus, the final form of the metric tensor is obtained as $G=\mathrm{diag}[1,\gamma_{4}^{2},0]$. Considering weak interactions between yarns and coating material, the specific forms of each individual nonlinear term/mode are given as

(5) $$\begin{array}{c} \;\psi^{\mathrm{iso}}=\left({\bar{I}}_{1}-3\right),\;\psi^{\mathrm{vol}}=\left({I_{3}}^{2}+{I_{3}}^{-2}-2\right),\\ \psi_{w}^{\mathrm{ti}}={\langle {\bar{J}}_{4w}-1\rangle }^{\gamma_{1}},\;\psi_{f}^{\mathrm{ti}}={\langle {\bar{J}}_{4f}-1\rangle }^{\gamma_{2}},\\ \psi^{\mathrm{int}}=\frac{1}{\left(\gamma_{3}+1\right)g^{\gamma_{3}}}\left[J_{4}^{\gamma_{3}+1}+J_{5}^{\gamma_{3}+1}-\ln I_{3}^{\left(\gamma_{3}+1\right)g^{\gamma_{3}}}-2g^{\gamma_{3}+1}\right],\;\mathrm{with}\;G=\mathrm{diag}\left[1,\gamma_{4}^{2},0\right]. \end{array}$$

In the above, the principal invariants of deformation tensor C are $I_{1}=\mathrm{tr}\left[C\right]$, $I_{2}=\mathrm{tr}\left[\mathrm{Cof}\left[C\right]\right]$ and $I_{3}=\det\left[C\right]$. In the transversely isotropic terms, the mixed invariants of C and $M_{i}$ are utilized; i.e., $J_{4w}=\mathrm{tr}\left[CM_{w}\right]$ and $J_{4f}=\mathrm{tr}\left[CM_{f}\right]$. Considering only tensile reinforcements, the Macaulay brackets are used to cancel out the transversely isotropic terms while the yarn direction is under compression; i.e., 〈(•)〉=[|•|+(•)]/2. The bar above the quantities implies the volume-preserving part of the invariants, i.e., ${\bar{I}}_{1}=I_{1}/I_{3}^{1/3}$ and ${\bar{J}}_{4i}=J_{4i}/I_{3}^{1/3}$. The mixed invariants in the interaction term are defined as $J_{4}=\mathrm{tr}\left[CG\right]$ and $J_{4}=\mathrm{tr}\left[\mathrm{Cof}\left[C\right]G\right]$ using the above described metric tensor. The trace of metric tensor is denoted as $g=\mathrm{tr}\left[G\right]=1+\gamma_{4}^{2}$.

### 4.3. Parameter Adjustment Procedure

As demonstrated in [15], the 4 model parameters $\gamma_{i}$ can be initially identified as a part of the model and kept fixed for each group of fabric material, i.e., either glass-PTFE or PES-PVC, also independent from their types, see Table 1. The main idea behind the identification of the model parameters is to minimize the deviation from the average value of the material parameters $\alpha_{i}$, which can be identified and computed for each individual stress–strain point [25]. Once this variation becomes minimized, the model parameters are identified and fixed for all types of a membrane material. In principle, the parameters $\gamma_{i}$ can also be individually identified to best match each membrane response by exploiting the full flexibility of the original formulation as in [14]. Nevertheless, the application of this method leads to the following advantages: (a) The model will be specialized for each group of membrane material and can be easily handled. (b) The number of total material parameters to be identified for each membrane is decreased to maximum 4 parameters, which is very similar to the simple linear elastic model; however, with a more accurate representation of membrane behavior [8]. (c) As it will be demonstrated, the identified model parameters can be used for all types of a fabric material at different stress–strain states, despite the fact that the material response is significantly changing from an initial load cycle (LC) to the latest one at LC 1000. (d) Following the method in [25], a unique identification of material parameters may become feasible as the remaining parameters appear only linearly in the formulation and thereby, the least-square error functional in terms of the stress deviation becomes convex in the material properties $\alpha_{i}$, see [8]. Moreover, since the material parameters mainly act as scaling factors of each deformation mode, a quite small sensitivity on the material behavior is automatically included. Therefore, to demonstrate the above mentioned points and show the generality of the nonlinear model, in this paper, the previously identified values of model parameters $\gamma_{i}$ from the authors’ work in [8,15,24] are directly implemented for the tested glass-PTFE and PES-PVC fabrics here. The values are listed in Table 3.

In the remainder of the paper, for simplicity of the formulations, the parameters in the linear elastic formulation ($E_{w},E_{f},\nu_{\mathrm{wf}},\nu_{\mathrm{fw}}$) and the material parameters in the nonlinear model ($\alpha^{\mathrm{iso}},\alpha_{w}^{\mathrm{ti}},\alpha_{f}^{\mathrm{ti}},\alpha^{\mathrm{int}}$) are all referred as material parameters $\alpha_{i}$. Furthermore, for a fair comparison between the two models, the material parameters $\alpha ={\left(\alpha_{1},\alpha_{2},\alpha_{3},\alpha_{4}\right)}^{T}$ of both models are fitted using the objective function defined

(6) $$L\left(\tilde{\alpha }\right)=\sum_{k}L_{k}\left(\tilde{\alpha }\right)\;\mathrm{with}\;L_{k}\left(\tilde{\alpha }\right)=\sqrt{\frac{1}{n_{\mathrm{mp}}}\sum_{i}^{n_{\mathrm{mp}}}{\left(\frac{{\left(•\right)}_{i}^{\mathrm{comp}}-{\left(•\right)}_{i}^{\exp}}{{\left(•\right)}_{i}^{\exp}}\right)}^{2}}.$$

The total error functional $L(\tilde{\alpha })$ is defined as the summation of different least-square error functions $L_{k}\left(\tilde{\alpha }\right)$, which are determined as the relative deviation of a computed value denoted as ${\left(•\right)}_{i}^{\mathrm{comp}}$ from its measured counterpart ${\left(•\right)}_{i}^{\exp}$. The $n_{\mathrm{mp}}$ shows the total number of measurement points. Subsequently, the material parameters are identified by minimization of the objective functional according to $\alpha =\;\mathrm{argmin}(L\left(\tilde{\alpha }\right))$. Note that during the optimization procedure, the measured engineering strains in the yarn directions, i.e., $\varepsilon_{w}$ and $\varepsilon_{f}$, are prescribed to the material model and the nominal stress components are computed. For the hyperelastic nonlinear material model, due to its 3-dimensional formulation, the third dimension deformation is computed by assuming an incompressible material response of woven fabrics. For both formulations, in the case of uniaxial tensile tests, the lateral strain was computed by iterating the transverse stress components to zero and thus, the deviation from the measured strain is included in the objective function.

For the further analysis in the next sections, a number of 10 load cycles out of the 1000 measured load cycles are selected and the material parameters are adjusted to each state, see Table 4 and Table 5. Five chosen load cycles belong to the first 100 cycles in order to highlight the change in the material parameters where the maximum portion of irreversible strain usually appears, i.e., LCs {4,5,10,20,75}. The other five are from the higher cycles where the material behavior is mainly elastic as the share of irreversible strain has decreased to a large extent, i.e., LCs {100, 250, 500, 750, 1000}. Table 4 shows the adjusted parameters of the nonlinear material model. Note that, while fitting the glass-PTFE materials using the nonlinear material model, it was observed that the isotropic parameter tends to become zero. This indicates that the isotropic response in the glass-PTFE material is generally non-dominant; therefore, the material behavior is mainly dominated by the yarns’ tensile stiffness and their direct crosswise interactions, cf. [8]. Thus, for the glass-PTFE materials, the parameter $\alpha^{\mathrm{iso}}$ is set to zero and the behavior is represented only with the remaining 3 parameters. This, however, was not observed in the case of PES-PVC where all parameters were influencing the material response. Table 5 shows the adjusted material parameters of the linear elastic formulation. The second Poisson’s ratio was computed based on Equation (3); however, the computed values are also included in Table 5.

Taking into account the change of adjusted parameters of both fabrics from LC 4 to LC 1000, see Table 4 and Table 5, it is noticeable that the material parameters directly linked to the material stiffness are remarkably increased. That is, in the case of the linear elastic model, the Young’s modulus $E_{w}$ and $E_{f}$ become almost doubled and in the case of the nonlinear material model, the two coefficients of transversely isotropic terms $\alpha_{w}^{\mathrm{ti}}$ and $\alpha_{f}^{\mathrm{ti}}$ are raised by a factor of 5. The interaction parameters $\alpha^{\mathrm{int}}$ of three tested fabrics also increase during cyclic loading, where, in the case of PES-PVC the values are much lower than the counterparts in glass-PTFE as the non-zero isotropic coefficient influences the material response. The Poisson’s ratios are all decreasing by advancing toward the LC 1000 indicating the change/decrease in the lateral contractions of the uniaxial tensile tests. Overall, the material response of the tested fabrics became stiffer after undergoing 1000 load cycles.

**Figure 3:**
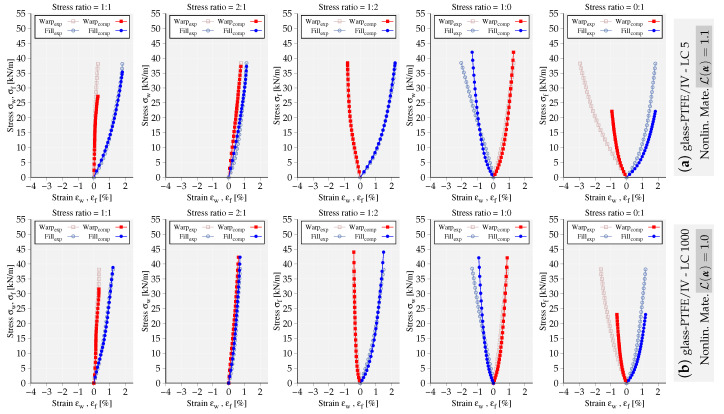
Mechanical response of the nonlinear material model fitting the stress–strain paths of glass-PTFE/IV (**a**) load cycle (LC) 5, (**b**) LC 1000. The obtained error value L(α) is reported next to each graph.

**Figure 4:**
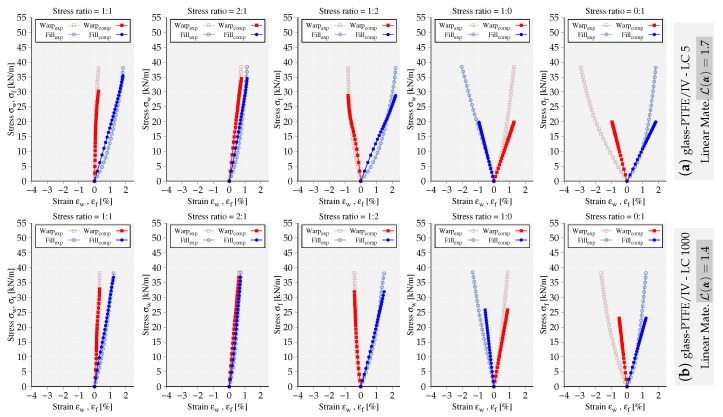
Mechanical response of the linear material model fitting the stress–strain paths of glass-PTFE/IV (**a**) LC 5, (**b**) LC 1000. The obtained error value L(α) is reported next to the graphs.

**Figure 5:**
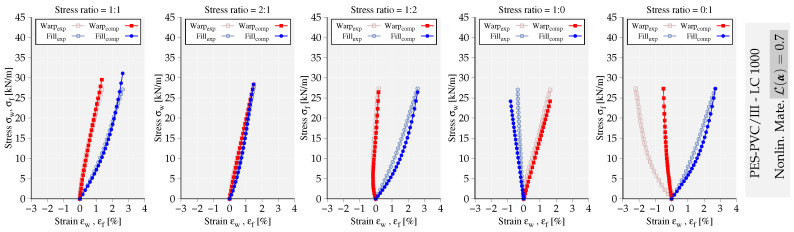
Mechanical response of the nonlinear material model fitting the stress–strain paths of PES-PVC/III, LC 1000. The obtained error value L(α) is reported next to graphs.

To exemplify the capability of the implemented models, the mechanical response of the fitted models to LC 5 and LC 1000 of glass-PTFE type IV are compared and illustrated in Figure 3 and Figure 4. The deviation of mechanical response from the experimental data is quantified using the value of objective functional $L(\tilde{\alpha })$, where the obtained values are reported next to each set of graphs. It is noteworthy that both models fit more accurately the stress–strain paths at LC 1000 with the obtained lower error values. In general, the nonlinear material model represents the experimental data of glass-PTFE with more accuracy, at least 40% lower error value compared to the linear elastic model. In Figure 5, the mechanical response of the nonlinear model fitted to LC 1000 of PES-PVC is shown. The nonlinear model is able to fit better both uniaxial stress–strain paths compared to the ones from glass-PTFE data. This becomes also evident from the overall error value which shows almost 30% less deviation from the considered experiments. More examples of PES-PVC data fitted by both formulations can be found in the previous work of the authors in [15].

## 5. Achieving the Saturated State by Extrapolation of Test Data from an Initial Cycle

Given the importance/relevance of considering the saturated elastic state, in this section, the evolution of material parameters at different load cycles is addressed. In fact, running a cyclic test for 1000 cycles becomes very expensive and not practical for industrial purposes. Therefore, taking into account the converging material response of woven fabrics after a large number of load cycles, it may be feasible to define a framework which facilitates the estimation of a material parameter for the latest cycles (e.g., LC 1000) or for some specifically desired load cycle from the parameter value adjusted to an initial state (e.g., LC 20). Consequently, one may avoid expensive experiments and still, within a good tolerance, estimate the parameter values at higher load cycles.

### 5.1. An Extrapolation Function to Estimate Parameter Values

In this section the focus is given to the characterization of an extrapolation function whose parameters are identified by fitting the function to the known material properties $\alpha_{i}$ of the two mathematical models at the chosen load cycles in Table 4 and Table 5. Thereby, it is expected that the proposed extrapolation function will be able to regenerate/estimate the material parameters, within an acceptable tolerance, at any state within e.g., 1000 load cycles. This is a similar procedure to the adjustment of material properties $\alpha_{i}$ described in Section 4.3 with the main difference that the proposed estimation function is fitted separately to independent behaviors. In the fitting procedure, the curve fitting application of Matlab [29] is used. The proposed estimation function is a simple power function with two coefficients *a* and *b*

(7) $$f\left(x\right)=ax^{b},$$

where the variable *x* indicates the LC index. The coefficients *a* and *b* are determined during the curve fitting procedure. Once *a* and *b* are found, the function f(x) provides an estimated value for the corresponding material property $\alpha_{i}$ by insertion of the load cycle index *x* as the only input; i.e., ${\hat{\alpha }}_{i,x}\equiv f\left(x\right)$. The hat above a quantity $\left(\hat{•}\right)$ indicates the estimated value. However, the ultimate goal of the proposed method is to keep the identified parameter *b* as a constant for a specific material property $\alpha_{i}$, i.e., $b_{\alpha_{i}}$, and recompute and replace the coefficient *a* from a known parameter value $\alpha_{i,20}$ at LC 20 from Table 4 and Table 5. Hence, the coefficient *a* may now be denoted as $a_{20,\alpha_{i}}$ and computed from $a_{20,\alpha_{i}}=\alpha_{i,20}/{\left(20\right)}^{b_{\alpha_{i}}}$. With the new $a_{20,\alpha_{i}}$ from LC 20, it should be possible to fairly estimate the material property at later load cycles considering the fact that, the computed constant $a_{20,\alpha_{i}}$ obtains usually a very close value to the fitted one $a_{\alpha_{i}}$. To better illustrate the method, the estimation of Young’s moduli in the warp direction at LC 1000 (x=1000) is carried out as

(8) $$\begin{array}{cc} {\hat{E}}_{w,x} & =a_{E_{w}}{\left(x\right)}^{b_{E_{w}}}\;\overset{\;\;\mathrm{determine}\;\left(a_{E_{w}},b_{E_{w}}\right)\;\;\;\;}{\leftarrow }\;\mathrm{curve}\;\mathrm{fitting}\;\mathrm{procedure}\\ a_{20,E_{w}} & =E_{w,20}/{\left(20\right)}^{b_{E_{w}}}\;\overset{\left(x=1000\right)}{\to }\;{\hat{E}}_{w,1000}=a_{20,E_{w}}{\left(1000\right)}^{b_{E_{w}}}. \end{array}$$

The choice of LC 20 over any lower load cycles, e.g., LC 5 or 10, was made since considering 20 load cycles is not expensive for experimental purposes and at the same time, the attained information from parameters at LC 5 might not be adequate to well estimate higher load cycles.

Taking into account the available experimental data of the two groups of fabrics, two different approaches are going to be applied here. First, considering the PES-PVC fabric where only one material type is available, with the help of the extrapolation function, the parameter values of the same material type will be estimated. With this, the accuracy of the proposed method in reproduction of the parameters at different load cycles will be investigated. Moreover, supplementary to the 10 considered LCs in Table 4 and Table 5, the material properties of 7 extra load cycles were additionally fitted for the PES-PVC and results are used in the extrapolation procedure. The extra load cycles are chosen among the higher LCs; i.e., 200, 300, 400, 600, 700, 800 and 900. In the second approach, since the data of two glass-PTFE types (III and IV) are available, the proposed method will be challenged by using the identified coefficients $b_{\mathrm{III}}$ of type III to recompute the coefficient $a_{\mathrm{IV}}$ and estimate the parameters of type IV and vice versa. In fact, it was observed that the obtained values of both glass-PTFE types evolved in a comparable manner during the 1000 load cycles; therefore, it should be possible to estimate the parameters within an acceptable tolerance. This also implies that for one kind of fabric material, e.g., PES-PVC, one constant *b* can be identified and implemented for all PES-PVC types and still the method estimates well the properties at higher load cycles provided that the material parameters for lower LC-numbers are available for the other material. In the second approach, only the data of 10 listed load cycles in Table 4 and Table 5 are considered.

Note that using the Matlab curve fitting tool, the coefficients *a* and *b* are adjusted by solving the minimization problem of $\min(\sum ||F\left(x_{i}\right)-y_{i}|{|}^{2})$ with the “Nonlinear Least Squares” method, where $F(x_{i})$ is a nonlinear function and $y_{i}$ is the material parameter value. Using the solver, the “Trust-Region” algorithm was applied, no weighting factors were used and no boundaries were defined for the coefficients. In the remainder of the paper, the subscripts of the coefficients $a_{\alpha_{i}}$ and $b_{\alpha_{i}}$ are dropped and the coefficients are shown as *a* and *b*, unless the presence of a subscript becomes necessary.

### 5.2. First Approach, Considering the Material Properties of PES-PVC

**Figure 6:**
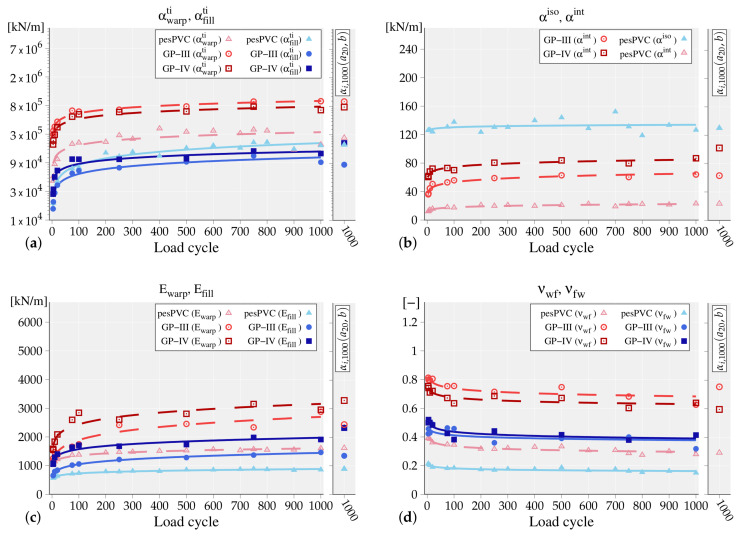
The material parameters (shown as dot) and the estimation function results (shown as dashed and solid lines) are illustrated. (**a**,**b**) The nonlinear material model parameters. (**c**,**d**) The linear elastic parameters. Next to each graph, the estimated parameters from the LC 20 data $\alpha_{i,1000}\left(a_{20},b\right)$ are additionally marked at LC 1000.

The introduced extrapolation function in Equation (7) is now tested in regeneration of the PES-PVC data followed by the second step in Equation (8). The first row of Table 4 and Table 5, i.e., the LC index, is inserted into the function and by adjustment of the parameters *a* and *b* using the curve fitting application [29], the function value f(x) becomes the closest possible to the material parameters $\alpha_{i}$ at the corresponding LC. Thus, associated to each material parameter $\alpha_{i}$, a pair of ${\left(a,b\right)}_{\alpha_{i}}$ is computed which are listed in the first two rows of Table 6. The computed pairs ${\left(a,b\right)}_{\alpha_{i}}$ in Matlab are generally accompanied with ±5% acceptable values; i.e., the parameters *a* and *b* are computed within their 95% trust region, and the reported values in Table 6 are the average of the computed lower and upper bounds. Note that, in the case of the linear elastic formulation, following the restriction of material symmetry, only one Poisson’s ratio $\nu_{\mathrm{wf}}$ was considered in the curve fitting and the second one, i.e., $\nu_{\mathrm{fw}}$, was evaluated from Equation (3). Since the main interest of this method is the estimation of parameter values at higher load cycles, LC 1000 is chosen as the central assessment value and hence only its corresponding material parameters are recomputed, see $\alpha_{i,1000}{\left(a,b\right)}_{\alpha_{i}}$ in the 3rd row of Table 6. Comparing the obtained estimated values to the last column of Table 4 and Table 5, the deviation from their original values is reported as a relative difference in the 4th row of Table 6. Except for one value of $\alpha_{w}^{\mathrm{ti}}$, all other parameters are estimated within an acceptable tolerance. By taking a closer look into the changes of the parameter $\alpha_{w}^{\mathrm{ti}}$ in Table 4, a large drop of the value from LC 750 to LC 1000 can be observed. Such radical changes, however, can be regulated by using the estimation function, as the function provides an averaged value by taking into account the course of parameter change within 1000 load cycles. This issue is, nevertheless, neglected here and the remaining computations are carried out with the obtained measurements. The obtained results using the computed values for ${\left(a,b\right)}_{\alpha_{i}}$ are illustrated in Figure 6. Considering the obtained graphs, it can be concluded that the simple power function appears to be able to well represent the parameters evolution with only two coefficients.

Subsequently, the second step is exerted which is recomputation and updating of the coefficient *a* for the identified and fixed values of *b* based on the known value of corresponding parameter at load cycle 20; i.e., the $\alpha_{i,20}$ listed in the 4th column of Table 4 and Table 5. The obtained values for the coefficients $a_{20,\alpha_{i}}$ are listed in Table 6. Comparing the computed $a_{20,\alpha_{i}}$ to the fitted ones in the first row, it is noticeable that the computed ones roughly lay in the acceptable ±5% region with only one exception $\alpha_{w}^{\mathrm{ti}}$ and thereby, it can be expected that the extrapolation from LC 20 will reproduce accurate data.

As the final step, the material properties at LC 1000, denoted as $\alpha_{i,1000}$ ($a_{20},b$), are computed with the replaced coefficient $a_{20,\alpha_{i}}$ and the fixed $b_{\alpha_{i}}$ and the estimated values are shown in the 6th row of Table 6. The acceptable deviation of $\alpha_{i,1000}$ ($a_{20},b$) from the originally fitted parameter values demonstrate the applicability of the proposed method. The estimated parameters at LC 1000 are shown in the small graphs next the original ones, see Figure 6.

### 5.3. Second Approach, Considering Two Types of Glass-PTFE Material Data

Considering the adjusted material parameters of two glass-PTFE types, the estimation function is independently fitted to the parameter values of each material model from the chosen load cycles, similar to the first step for PES-PVC. The obtained coefficients of the extrapolation function are reported in the first two rows of Table 7 and Table 8 for glass-PTFE types III and type IV, respectively. By employment of the fitted coefficients to the extrapolation function, the material parameters of the same type are estimated and the deviation of the extrapolated values from the original ones, see Table 4 and Table 5, are reported in the fourth row of Table 7 and Table 8. The estimated material parameters of both glass-PTFE fabrics are within an acceptable range where the maximum deviations from the original values are observed for $\alpha_{f}^{\mathrm{ti}}$ of glass-PTFE III with almost 20%. As it was also mentioned in the last section, such large deviations are not avoidable during the experiments/measurements of 1000 load cycles. Nevertheless, the estimation function would provide an average value at each LC considering the parameter change during 1000 load cycles. Using the obtained coefficients, the trend line of each estimated material parameter is depicted in Figure 6.

As it was previously mentioned, in order to demonstrate the feasibility of the proposed method, the coefficients *b* of two glass-PTFE types will be swapped. Therefore, the found constants of type III, $b_{\mathrm{III}}$, are used to recompute the coefficients $a_{\mathrm{IV}}$ of type IV, i.e., $a_{20,\alpha_{i}}\left(b_{\mathrm{IV}},\alpha_{i,20}\right)$, and vice versa. The obtained values of coefficients $a_{20,\alpha_{i}}\left(b_{\mathrm{IV}},\alpha_{i,20}\right)$ and $a_{20,\alpha_{i}}\left(b_{\mathrm{III}},\alpha_{i,20}\right)$ are reported in Table 7 and Table 8, respectively. Subsequently, with the computed coefficients, the associated material parameters are estimated at LC 1000. The extrapolated material parameters of types III and IV shown as $\alpha_{i,1000}\left(a_{20},b_{\mathrm{IV}}\right)$ and $\alpha_{i,1000}\left(a_{20},b_{\mathrm{III}}\right)$ are listed in Table 7 and Table 8. Taking into account the deviation values, the overall estimation results are of a lower quality; however, in the same manner as it was estimated before. That is, if a parameter was originally estimated smaller than the original values, using the swapped coefficients, the parameters are again estimated smaller than the original ones. This was expected as the same coefficients *b* of types III and IV generally have similar values; i.e., same material parameters of two types evolve analogously. Considering the estimated parameters of the nonlinear material model for type III, the estimated parameters became in fact closer to the original values. The obtained values of the material parameters from the swapped coefficients are marked at LC 1000 next to each graph. In the following section, the accuracy of the proposed extrapolation method will be tested/analyzed in the simulation of a roof structure, using the estimated and the originally adjusted material parameters.

## 6. Impact of Saturated Elastic State Properties on Structural Response

In this section, considering a complex structural boundary value problem, the impact of the saturated elastic state properties on the structural response of fabric models is analyzed. To this end, the structural behavior of the adjusted models at 3 states, i.e., LC 5, LC 20 and LC 1000, will be compared. These 3 different states are intentionally chosen in order to underline the effect of saturation by comparing the commonly used “elastic state” in industrial applications, i.e., LC 5, to the latest saturated elastic state in our study, i.e., LC 1000. The choice of LC 20 will be used as a testing tool to demonstrate the change of state from LC 5 to LC 20 and from LC 20 to the latest cycle at LC 1000. Naturally, the extrapolation method will also be assessed by employing the estimated parameters of LC 1000 in the structural example.

The structural boundary value problem is a roof structure similar to the one considered in [8]. The roof is initially curved on the sides and it has a real-size geometry. The warp yarns, following the curvature of the roof, are located such that they connect the upper ring to the bottom edges. The fill yarns are located perpendicular to the warp direction. The upper ring has a diameter of 600 mm. The maximum distance between the legs is 4000 mm and height is 1500 mm. Due to the complex geometry and the applied boundary conditions, it is expected that different variations of stress ratio, including the ones in the experimental tests, may occur within the structure. Following the natural installation procedure of tension fabrics, i.e., pre-tensioning of membranes, the upper ring is pulled in the Z-direction to activate prestress. Over the whole simulation, the roof is loaded with gravity force, where the density of the roof is set to $\rho =1\times 10^{-6}\;\mathrm{kg}/{\mathrm{mm}}^{3}$. To resemble an everyday loading condition, the roof is loaded with a static wind force $F_{\mathrm{wind}}$ = 2.4 $\mathrm{kN}/m^{2}$. The bottom edges of the structure is fully clamped. The roof is discretized using 4 node square elements, “Quad” element in Abaqus, with the maximum size of 40 mm. The chosen element type is S4R shell element which may allow for finite strains. For the material description, the phenomenological models described in Section 4 was implemented in terms of the user material (UMAT) interface. For more details on this example, one may refer to [8]. Note that at this stage, the numerical example neglects variations of prestress in the structure due to irreversible strain in the different investigated load cases. Thus, the calculated stress and deflection do not properly reflect realistic stress and deflection of the investigated structure under the investigated load. The intention of the numerical example is rather to emphasize the impact of material stiffness parameters taken from the range between those of early and late load cycles. The interaction with prestress variations will be investigated in future research. Note that here we only consider pressure, no suction, although suction may be the more relevant loading. Thus, the simulation should be interpreted rather as an example showing a potential influence of different material parameters on a realistic scenario.

**Figure 7:**
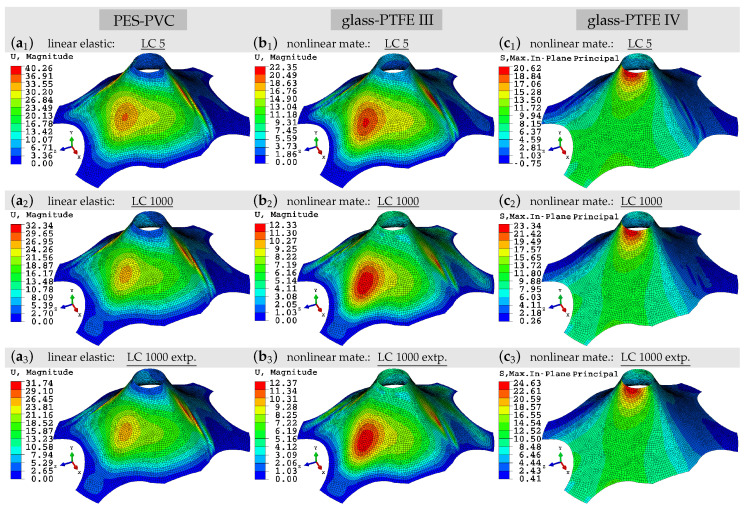
Roof structure: comparison of material models response at different states. (**a**${}_{1-3}$) Displacement magnitude of the PES-PVC material at LC 5, LC 1000 and LC 1000 extrapolated. (**b**${}_{1-3}$) Displacement magnitude of the glass-PTFE III material at LC 5, LC 1000 and the extrapolated state of LC 1000 using the coefficients of type IV. (**c**${}_{1-3}$) Maximum in-plane Cauchy stress of the glass-PTFE IV material at LC 5, LC 1000 and the extrapolated state of LC 1000 using the coefficients of type III.

In Figure 7, the structural response of the material models under the described boundary conditions, using PES-PVC and glass-PTFE types III and IV properties, are illustrated in extracts. Figure 7a${}_{1}$,a${}_{2}$ respectively show the obtained displacement field of PES-PVC using the linear elastic model at LC 5 and LC 1000. Taking into account both fields, the maximum displacement magnitude recorded at LC 5 decreases by 20% compared to the result of LC 1000. This was in fact expected, since the associated adjusted stiffness parameters became almost doubled, see Table 5. Using the nonlinear material model, a similar behavior is observed with almost 30% decrease of the maximum displacement. The obtained results of both formulations are listed in Table 9.

As the fabric material becomes stiffer during the applied 1000 cycles, the obtained stress values are also increasing, see Table 9; however, with a moderate change of 5–14% from LC 5 to LC 1000. Considering the obtained results for LC 20 also support the statement that the material response from LC 5 to LC 20 changes quickly, the slope of this alternation significantly decreases while going toward LC 1000. That is, the more one proceeds in the number of load cycles, the less the variation in the material properties.

In addition, the roof structure was simulated using the extrapolated parameters of PES-PVC from Table 6. Although, the estimation values in some cases deviated by 30% from the original ones, in the structural response this deviation becomes negligible. The maximum obtained stress and displacement values from the simulation using the extrapolated parameters are listed under “LC 1000 extp.” in Table 9. To quantify the accuracy, the deviation is not more than 2% compared to the responses obtained with original parameters, see “LC 1000” values in Table 9. The displacement field attained by the estimated parameter of linear elastic model for PES-PVC is shown in Figure 7a${}_{3}$.

Subsequently, the same simulations are carried out using the parameters of two types of glass-PTFEs. To show some of the obtained results, using the nonlinear material model, the displacement fields of type III and Cauchy stress fields of type IV are depicted in Figure 7b,c, respectively. The maximum displacements obtained in both simulations become almost halved going from LC 5 to LC 1000. Similar to PES-PVC results, the change in maximum stress values is generally lower compared to change of maximum displacements. The extracted maximum stress and displacement values of linear elastic and nonlinear material formulations are reported in Table 10.

As before, the simulations are carried out with the extrapolated data of LC 1000 for both glass-PTFE types. Note that for the extrapolation of the material parameters of type IV, the coefficient $b_{\mathrm{III}}$ was employed, and similarly, for parameter estimation of type III, the coefficient *b* of type IV was used as described in Section 5.3. The obtained results are illustrated in the Figure 7b${}_{3}$,c${}_{3}$. Comparing the extracted values in Table 10 under “LC 1000 extp.” to the ones listed under “LC 1000”, it is demonstrated that the estimation function was able to reproduce material parameters fair enough, such that, the structural response is marginally affected.

Therefore, it can be concluded that, if in general the estimated parameters for LC 1000 obtained from LC 20 moderately deviate from the originally adjusted ones at LC 1000, the estimation function can sufficiently extrapolate the parameters such that the qualitative and quantitative structural responses are fairly accurate and the original parameter deviation becomes negligible, at least for the investigated structure. Furthermore, the results obtained for both glass-PTFE types demonstrated that for different types of a membrane material, it is actually practical to use similar coefficients $b_{\alpha_{i}}$ in the estimation function for all types. That is, the coefficients of estimation function are not only able to extrapolate the parameters of the same membrane, as in the case of PES-PVC, but they can also be employed in the extrapolation of other types of a material kind, as in the case of glass-PTFE. The general applicability also on other structural types has to be shown in future investigations.

Another observation made from Table 9 and Table 10 is that the use of the linear approach is mostly safe-sided for the investigated structure, either regarding computed stresses or displacements. Both are larger than resulting from the nonlinear approach, with stresses for glass-PTFE type III as the only exception. Particularly, stresses and displacements from the “linear material” LC 5 are larger than those from the “nonlinear material” LC 1000. From a practical point of view, this indicates that the linear approach is a less accurate however a safer choice, even with parameters of LC 5.

## 7. Conclusions

The mechanical saturation behavior of two types of PTFE-coated glass fiber fabrics was experimentally investigated. Furthermore, an extrapolation function was derived enabling an estimation of late cycle stiffness parameters from early cycle test data and the impact of late cycle stiffness parameters compared to early cycle ones was illustrated by a numerical example. The saturation behaviors of both types III and IV of glass-PTFE fabrics were found to be similar to each other and to a recently reported PES-PVC fabric. The irreversible strain increments decrease fast in the first load cycles and settle down to low constant levels with low scatter around it—although compared to the PES-PVC fabric, bigger scatter and higher mean values were observed.

Based on the applied saturation criteria, this inspection characteristic saturates after 10 ± 4 full service load cycles. The other both saturation inspection characteristics checked, being the total strain increment and the intensity of nonlinearity, oftentimes require many thousands of load cycles to appropriately approach the saturated state. Considerably higher intensities of nonlinearity were observed for glass fiber fabrics compared to PES-PVC, particularly in the stress–strain paths of the fill direction. Overall, both investigated fabrics show a saturating mechanical behavior approaching the elastic share of the initially inelastic stress–strain paths while converting them into an elastic state. Changes of stiffness parameters in late cycles are slow but can be considerably compared to early ones. For instance, elastic moduli in load cycle 1000 can be roughly twice as high as the ones of load cycle 5.

It is not reasonable in industrial projects to perform cyclic tests with many thousands of load cycles to find out the saturated state of a fabric. Thus, the second goal of the present work was to establish an extrapolation function that allows to predict the stiffness behavior of late cycles from test data of early cycles. An easy to handle power function with only two coefficients to be identified was developed and validated with test data of the earlier reported PES-PVC fabric. Based on test data of load cycle 20, it allows the prediction of specific stiffness parameters with good accuracy. Actually, higher deviations of up to 30% appear in single situations but become negligible in structural analyses as was shown in a numerical example. The functionality was shown for the widely used orthotropic linear elastic material model as well as for a recently developed hyperelastic anisotropic material model. Furthermore, it was validated with the two tested types of glass-PTFE fabrics. It could be shown that a good prediction is possible for the tested 1000 load cycles, even if coefficients for type III and IV were swapped, i.e., the function is capable to predict material parameters of a type IV with test data of type III and vice versa. This gives rise to the hope that coefficients once identified can be applied to a whole kind of materials, i.e., for instance for all types of glass-PTFE.

In the last section, stiffness parameters of different states of all considered materials were used as input in a complex structural boundary value problem. Both linear and nonlinear material models were considered. Moreover, stiffness parameters originally adjusted to the test data were used as well as extrapolated ones. It was found that the impact of late cycle parameters can be significant. Comparing load cycles 5 and 1000, stress differences were up to approximately 30% and differences in deformation could be up to roughly 50%. Using parameters achieved from the extrapolation brought deviations of not more than 2% compared to responses obtained with original parameters. Therefore, it can be concluded that, if in general the estimated parameters for LC 1000 obtained from LC 20 moderately deviate from the originally adjusted ones at LC 1000, the estimation function can sufficiently extrapolate the parameters such that the qualitative and quantitative structural responses are fairly accurate and the original parameter deviation becomes negligible.

Overall, the high impact shows the importance of a correct handling of stiffness parameters. The orthotropic linear material model turned out to be—besides being less accurate—safe-sided when using early load cycle stiffness parameters, either considering computed stresses or displacements. However, if the saturated elastic response is to be precisely investigated it appears to be not sufficient to use the parameters from LC 5. Instead the saturated state load cycle should be used—even if only extrapolated. It is expected that with the extrapolation function even load cycles higher than 1000 may be analyzed. It is a topic of future research to validate this. Knowledge about the elastic share of the stress–strain paths of woven fabrics facilitates the further development of elastic and visco-elastoplastic material models. The presented saturation analysis and the developed extrapolation function will serve these demanded advancements. Together with stiffness variations, prestress variations have to be considered in the structural analysis. Future research in simplified elastic modeling should properly take this aspect into account and develop a structural analysis guideline consistent with the boundary conditions in membrane structure analysis, integrating formfound geometry and the correlated prestress state as the analysis’ foundation. For future viscoelastic material models, it is recommended to investigate the impact of coating thickness on viscoelastic effects.

## Figures and Tables

**Table 1:** Basic properties of the investigated fabrics.

Material	Property	Standard/Reference	Specified Values	Unit
**N${}^{\circ }$1: Glass-PTFE**	**Tensile strength $f_{t}$ (warp/fill)**	DIN EN ISO 1421	140/120	kN/m
	**Type**	1)	III	-
	**Weave**	-	L 1/1	-
	**Total weight**	DIN EN ISO 2286-2	1150	$g/m^{2}$
**N${}^{\circ }$2: Glass-PTFE**	**Tensile strength $f_{t}$ (warp/fill)**	DIN EN ISO 1421	160/160	kN/m
	**Type**	1)	IV	-
	**Weave**	-	L 1/1	-
	**Total weight**	DIN EN ISO 2286-2	1600	$g/m^{2}$
**PES-PVC**	**Tensile strength $f_{t}$ (warp/fill)**	DIN EN ISO 1421	130/114	kN/m
	**Type**	1)	III	-
	**Weave**	-	P 1/1	-
	**Total weight**	DIN EN ISO 2286-2	1200	$g/m^{2}$

1) According to the proposed harmonization in [7].

**Table 2:** Saturated state load cycles for the investigated fabrics.

Material			Computed Saturated State Load Cycle * for		
	Stress Ratio	Principal	Irreversible	Total	Intensity of
	Warp:Fill	Direction	Strain Inc.	Strain Inc.	Nonlinearity
**N${}^{\circ }$1: Glass-PTFE Type III**	1:1	1 (warp)	4	17,999	8,378,928
		2 (fill)	9	16,161	968,343
	2:1	1 (warp)	11	50	432,654
		2 (fill)	10	27,318	6230
	1:2	1 (fill)	14	18,193	51,811
		2 (warp)	12	104,124	5183
	1:0	1 (warp)	9	10,920	20,472
		2 (fill)	10	340	6879
	0:1	1 (fill)	9	30,893	75,419
		2 (warp)	12	5093	50
**N${}^{\circ }$2: Glass-PTFE Type IV**	1:1	1 (warp)	17	12,842	240,938
		2 (fill)	10	21,041	387,476
	2:1	1 (warp)	4	772	23,904
		2 (fill)	7	10,723	1156
	1:2	1 (fill)	10	3429	171
		2 (warp)	8	62	12,784
	1:0	1 (warp)	5	3269	1916
		2 (fill)	15	5462	31,348
	0:1	1 (fill)	15	51	2223
		2 (warp)	11	7740	22,608

* Achieved per definition for irreversible strain increment when first time lower than 1% of the total strain increment and for total strain increment and intensity of nonlinearity when deviation from the asymptote of the fitted asymptotic function first time lower than 3%.

**Table 3:** The previously identified model parameters for glass-PTFE and PES-PVC fabrics.

Model Parameters	$\gamma_{1}$[-]	$\gamma_{2}$[-]	$\gamma_{3}$[-]	$\gamma_{4}$[-]
**PES-PVC**	5	5	5	0.40
**glass-PTFE**	4	4	6	0.81

**Table 4:** Adjusted material parameters of the nonlinear material model.

Glass-PTFE III										
	**LC 4**	**LC 5**	**LC 10**	**LC 20**	**LC 75**	**LC 100**	**LC 250**	**LC 500**	**LC 750**	**LC 1000**
$\alpha^{\mathrm{int}}$ [kN/m]	36	37.2	44.5	50.7	53	56	59	63	60.3	64
$\alpha_{w}^{\mathrm{ti}}$ [kN/m]	266,570	30,7124	359,411	435,829	665,622	642,799	690,680	784,072	945,345	955,646
$\alpha_{w}^{\mathrm{ti}}$ [kN/m]	15,389	20,108	28,189	38,094	60,114	66,605	74,520	93,432	117,931	91,996
**Glass-PTFE IV**										
$\alpha^{\mathrm{int}}$ [kN/m]	60.4	61.8	68.2	72.2	73.1	70.2	80.5	83.8	79.7	86.7
$\alpha_{w}^{\mathrm{ti}}$ [kN/m]	181,801	211,185	263,986	352,784	529,469	579,013	633,006	646,296	777,117	679,436
$\alpha_{w}^{\mathrm{ti}}$ [kN/m]	27,085	32,607	52,255	66,909	103,519	103,297	103,013	105,214	141,614	128,668
**PES-PVC**										
$\alpha^{\mathrm{iso}}$ [kN/m]	126	126	126.5	124	130.6	137.6	130	144	131.1	126.4
$\alpha^{\mathrm{int}}$ [kN/m]	12	12.7	14.2	15.7	17.8	17.3	20	21	22.3	23
$\alpha_{w}^{\mathrm{ti}}$ [kN/m]	44,986	53,267	85,084	102,810	185,040	194,340	257,940	294,920	321,230	181,580
$\alpha_{w}^{\mathrm{ti}}$ [kN/m]	16,368	19,101	30,543	44,276	71,649	74,725	114,390	158,460	196,220	174,260

**Table 5:** Adjusted material parameter of the linear elastic formulation.

Glass-PTFE III										
	**LC 4**	**LC 5**	**LC 10**	**LC 20**	**LC 75**	**LC 100**	**LC 250**	**LC 500**	**LC 750**	**LC 1000**
$E_{w}$ [kN/m]	1252	1166.2	1470.8	1382.5	1656.8	1743.5	2414.3	2452.5	2335.1	2877.8
$E_{f}$ [kN/m]	650.6	666.5	792.7	839	1016.4	1057.5	1214.2	1279.8	1367.7	1461.6
$\nu_{\mathrm{wf}}$ [-]	0.81	0.80	0.79	0.81	0.75	0.76	0.71	0.75	0.68	0.62
$\nu_{\mathrm{fw}}$ [-]	0.42	0.46	0.43	0.49	0.46	0.46	0.36	0.39	0.40	0.32
**Glass-PTFE IV**										
$E_{w}$ [kN/m]	1586.3	1548.6	1833.8	2087.6	2595.2	2842.1	2605.2	2806.8	3148.1	2948.7
$E_{f}$ [kN/m]	1049.2	1088	1292.2	1400.1	1640.2	1708.7	1676.4	1735.7	1980.8	1909.6
$\nu_{\mathrm{wf}}$ [-]	0.75	0.74	0.71	0.72	0.67	0.63	0.69	0.67	0.60	0.64
$\nu_{\mathrm{fw}}$ [-]	0.50	0.52	0.50	0.48	0.42	0.38	0.44	0.42	0.38	0.41
**PES-PVC**										
$E_{w}$ [kN/m]	1031	1059	1142	1217.2	1367.5	1376.7	1470.3	1524.5	1569	1598.9
$E_{f}$ [kN/m]	562	563.1	598.3	636.3	711.6	732	785.2	852.9	886.6	854.3
$\nu_{\mathrm{wf}}$ [-]	0.39	0.39	0.38	0.36	0.35	0.34	0.32	0.33	0.29	0.28
$\nu_{\mathrm{fw}}$ [-]	0.21	0.21	0.20	0.19	0.18	0.18	0.17	0.19	0.16	0.15

**Table 6:** Extrapolated material parameters and the computed coefficients of the estimation function using PES-PVC data. Deviation is computed as relative difference of an estimated parameter from its original value in Table 4 and Table 5.

PES-PVC							
$f\left(x\right)=ax^{b}$	$\alpha^{\mathrm{iso}}$ [kN/m]	$\alpha^{\mathrm{int}}$ [kN/m]	$\alpha_{w}^{\mathrm{ti}}$ [kN/m]	$\alpha_{f}^{\mathrm{ti}}$ [kN/m]	$E_{w}$ [kN/m]	$E_{f}$ [kN/m]	$\nu_{\mathrm{wf}}$ [-]
*a*[±5%]	124	11.2	65,440	15,630	967	500	0.431
*b*[±5%]	0.012	0.102	0.216	0.363	0.073	0.082	−0.055
$\alpha_{i,1000}{\left(a,b\right)}_{\alpha_{i}}$	134	22.6	291,570	191,716	1602	881	0.295
Deviation[%]	5.8	−1.9	60.5	10	0.2	3.1	6.2
$a_{20,\alpha_{i}}$ ($b,\alpha_{i,20}$)	120	11.6	53,780	14,929	977	498	0.425
$\alpha_{i,1000}$ ($a_{20},b$)	130	23.3	239,620	183,115	1620.5	877	0.291
Deviation[%]	2.4	1.4	31	5	1.3	2.6	4.6

**Table 7:** Extrapolated material parameters and the computed coefficients of the estimation function using glass-PTFE III data. Deviation is computed as relative difference of an estimated parameter from its original value in Table 4 and Table 5.

Glass-PTFE Type III							
$f\left(x\right)=ax^{b}$	$\alpha^{\mathrm{iso}}$ [kN/m]	$\alpha^{\mathrm{int}}$ [kN/m]	$\alpha_{w}^{\mathrm{ti}}$ [kN/m]	$\alpha_{f}^{\mathrm{ti}}$ [kN/m]	$E_{w}$ [kN/m]	$E_{f}$ [kN/m]	$\nu_{\mathrm{wf}}$ [-]
$a_{\mathrm{III}}$ [±5%]	-	35.3	234,800	16,600	931	553	0.863
$b_{\mathrm{III}}$ [±5%]	-	0.089	0.205	0.275	0.154	0.139	−0.034
$\alpha_{i,1000}$($a_{\mathrm{III}},b_{\mathrm{III}}$)	-	65.3	966,937	110,562	2697	1444	0.682
Deviation[%]	-	2.3	1.2	20.2	−6.2	−1.2	10
$a_{20,\alpha_{i}}$ ($b_{\mathrm{IV}},\alpha_{i,20}$)	-	43	236,540	20,533	894${}^{*}$	582${}^{*}$	0.847${}^{*}$
$\alpha_{i,1000}$ ($a_{20},b_{\mathrm{IV}}$)	-	63	968,067	85,380	2442.7${}^{*}$	1350.6${}^{*}$	0.75${}^{*}$
Deviation[%]	-	−1.5	1.2	−7.2	−15	−7.5	20

${}^{*}$ Computed using $b_{\mathrm{IV}}\left[+5\%\right]$

**Table 8:** Extrapolated material parameters and the computed coefficients of the estimation function using glass-PTFE IV data. Deviation is computed as relative difference of an estimated parameter from its original value in Table 4 and Table 5.

Glass-PTFE Type IV							
$f\left(x\right)=ax^{b}$	$\alpha^{\mathrm{iso}}$ [kN/m]	$\alpha^{\mathrm{int}}$ [kN/m]	$\alpha_{w}^{\mathrm{ti}}$ [kN/m]	$\alpha_{f}^{\mathrm{ti}}$ [kN/m]	$E_{w}$ [kN/m]	$E_{f}$ [kN/m]	$\nu_{\mathrm{wf}}$ [-]
$a_{\mathrm{IV}}$[±5%]	-	57.8	189,900	33,430	1464	1005	0.776
$b_{\mathrm{IV}}$[±5%]	-	0.056	0.204	0.206	0.111	0.097	−0.03
$\alpha_{i,1000}$($a_{\mathrm{IV}},b_{\mathrm{IV}}$)	-	85	777,186	139,007	3152	1964	0.63
Deviation[%]	-	−2.1	14.4	8	6.8	2.8	−1.5
$a_{20,\alpha_{i}}$ ($b_{\mathrm{III}},\alpha_{i,20}$)	-	55.3	190,953	29,400	1475${}^{*}$	949${}^{*}$	0.833${}^{*}$
$\alpha_{i,1000}$ ($a_{20},b_{\mathrm{III}}$)	-	102	786,370	195,819	3286.5${}^{*}$	2325.5${}^{*}$	0.59${}^{*}$
Deviation[%]	-	18	15.7	52	11.45	21.8	−6.7

${}^{*}$ Computed using $b_{\mathrm{III}}\left[-5\%\right]$

**Table 9:** The obtained maximum displacement and in-plane Cauchy stress values in simulation of the roof structure. The material is the PES-PVC III with adjusted and extrapolated parameters.

PES-PVC								
	Displacement Magnitude [mm]				Stress, max. In-Plane [MPa]			
	**LC 5**	**LC 20**	**LC 1000**	**LC 1000 extp.**	**LC 5**	**LC 20**	**LC 1000**	**LC 1000 extp.**
Linear Material	40.3	37.6	32.3	31.7	19.7	20.6	22.5	22.6
Nonlinear Mate.	36.7	32.2	25.9	25.4	17.6	17.9	18.5	18.6

**Table 10:** The obtained maximum displacement and in-plane Cauchy stress values in simulation of the roof structure. The materials are the glass-PTFE types III and IV with the adjusted and extrapolated parameters.

Glass-PTFE III								
	Displacement Magnitude [mm]				Stress, max. In-Plane [MPa]			
	**LC 5**	**LC 20**	**LC 1000**	**LC 1000 extp.**	**LC 5**	**LC 20**	**LC 1000**	**LC 1000 extp.**
Linear Material	29.2	25.2	14.3	15.7	22	23.5	29.1	28.3
Nonlinear Mate.	22.4	17.6	12.3	12.4	21.7	22.1	23.9	23.9
**Glass-PTFE IV**								
Linear Material	23.2	17.9	13.8	12.8	24.3	26.8	30.4	31.9
Nonlinear Mate.	17.5	14.5	11.4	10.2	20.6	21.6	23.3	24.6

## Supplementary Materials

The following are available online at https://www.mdpi.com/1996-1944/13/19/4243/s1, Figure S1: Curves of the saturation inspection characteristics for stress ratio 1:1, principal direction. (a) Irreversible strain increment vs. load cycles; (b) irreversible strain increment vs. load cycles; (c) total strain vs. load cycles; (d) total strain vs. load cycles; (e) intensity of nonlinearity vs. load cycles and (f) intensity of nonlinearity vs. load cycles, Figure S2: Curves of the saturation inspection characteristics for stress ratio 1:1, transverse direction. (a) Irreversible strain increment vs. load cycles; (b) irreversible strain increment vs. load cycles; (c) total strain vs. load cycles; (d) total strain vs. load cycles; (e) intensity of nonlinearity vs. load cycles and (f) intensity of nonlinearity vs. load cycles, Figure S3. Curves of the saturation inspection characteristics for stress ratio 2:1, principal direction. (a) Irreversible strain increment vs. load cycles; (b) irreversible strain increment vs. load cycles; (c) total strain vs. load cycles; (d) total strain vs. load cycles; (e) intensity of nonlinearity vs. load cycles and (f) intensity of nonlinearity vs. load cycles, Figure S4. Curves of the saturation inspection characteristics for stress ratio 2:1, transverse direction. (a) Irreversible strain increment vs. load cycles; (b) irreversible strain increment vs. load cycles; (c) total strain vs. load cycles; (d) total strain vs. load cycles; (e) intensity of nonlinearity vs. load cycles and (f) intensity of nonlinearity vs. load cycles, Figure S5. Curves of the saturation inspection characteristics for stress ratio 1:2, principal direction. (a) Irreversible strain increment vs. load cycles; (b) irreversible strain increment vs. load cycles; (c) total strain vs. load cycles; (d) total strain vs. load cycles; (e) intensity of nonlinearity vs. load cycles and (f) intensity of nonlinearity vs. load cycles, Figure S6. Curves of the saturation inspection characteristics for stress ratio 1:2, transverse direction.(a) Irreversible strain increment vs. load cycles; (b) irreversible strain increment vs. load cycles; (c) total strain vs. load cycles; (d) total strain vs. load cycles; (e) intensity of nonlinearity vs. load cycles and (f) intensity of nonlinearity vs. load cycles, Figure S7. Curves of the saturation inspection characteristics for stress ratio 1:0, principal direction. (a) Irreversible strain increment vs. load cycles; (b) irreversible strain increment vs. load cycles; (c) total strain vs. load cycles; (d) total strain vs. load cycles; (e) intensity of nonlinearity vs. load cycles and (f) intensity of nonlinearity vs. load cycles, Figure S8. Curves of the saturation inspection characteristics for stress ratio 1:0, transverse direction. (a) Irreversible strain increment vs. load cycles; (b) irreversible strain increment vs. load cycles; (c) total strain vs. load cycles; (d) total strain vs. load cycles; (e) intensity of nonlinearity vs. load cycles and (f) intensity of nonlinearity vs. load cycles, Figure S9. Curves of the saturation inspection characteristics for stress ratio 0:1, principal direction. (a) Irreversible strain increment vs. load cycles; (b) irreversible strain increment vs. load cycles; (c) total strain vs. load cycles; (d) total strain vs. load cycles; (e) intensity of nonlinearity vs. load cycles and (f) intensity of nonlinearity vs. load cycles, Figure S10. Curves of the saturation inspection characteristics for stress ratio 0:1, transverse direction. ((a) Irreversible strain increment vs. load cycles; (b) irreversible strain increment vs. load cycles; (c) total strain vs. load cycles; (d) total strain vs. load cycles; (e) intensity of nonlinearity vs. load cycles and (f) intensity of nonlinearity vs. load cycles.
