# Supplementary material for: The Elastic Share of Inelastic Stress–Strain Paths of Woven Fabrics
Source: Materials (Basel). 2020 Sep 23;13(19):4243. doi: 10.3390/ma13194243 (PMC7579112; doi:10.3390/ma13194243)
Supplement: Supplementary file 1 [file materials-13-04243-s001.pdf]

materials

Supplementary Materials

The elastic share of inelastic stress-strain paths of woven fabrics

Mehran Motevalli [1,†], Jörg Uhlemann [2,‡], Natalie Stranghöner [2,‡] and Daniel Balzani [1,†,*]

1 Chair of Continuum Mechanics, Ruhr-University Bochum, D-44801 Bochum, Germany; mehran.motevalli@rub.de
2 Institute for Metal and Lightweight Structures, University of Duisburg-Essen, 45117 Essen, Germany; joerg.uhlemann@uni-due.de (J.U.); natalie.stranghoener@uni-due.de (N.S.)
* Correspondence: daniel.balzani@rub.de; Tel.: +49-234-32-23080
† These authors contributed equally to this work.
‡ These authors contributed equally to this work.

Received: 26 August 2020; Accepted: 17 September 2020; Published: 23 September 2020

In addition of section 3 of the paper, this document provides all curve progressions of all three saturation inspection characteristics for both investigated glass-PTFE fabrics type III and IV, see Figures S1 to S10. Each Figure summarizes the diagrams in principal or transverse direction, respectively, for one of the five tested stress ratios. Note that principal direction is always the direction which is subjected to the major stress of a stress ratio. For stress ratio 1:1, principal direction equals warp direction per definition.

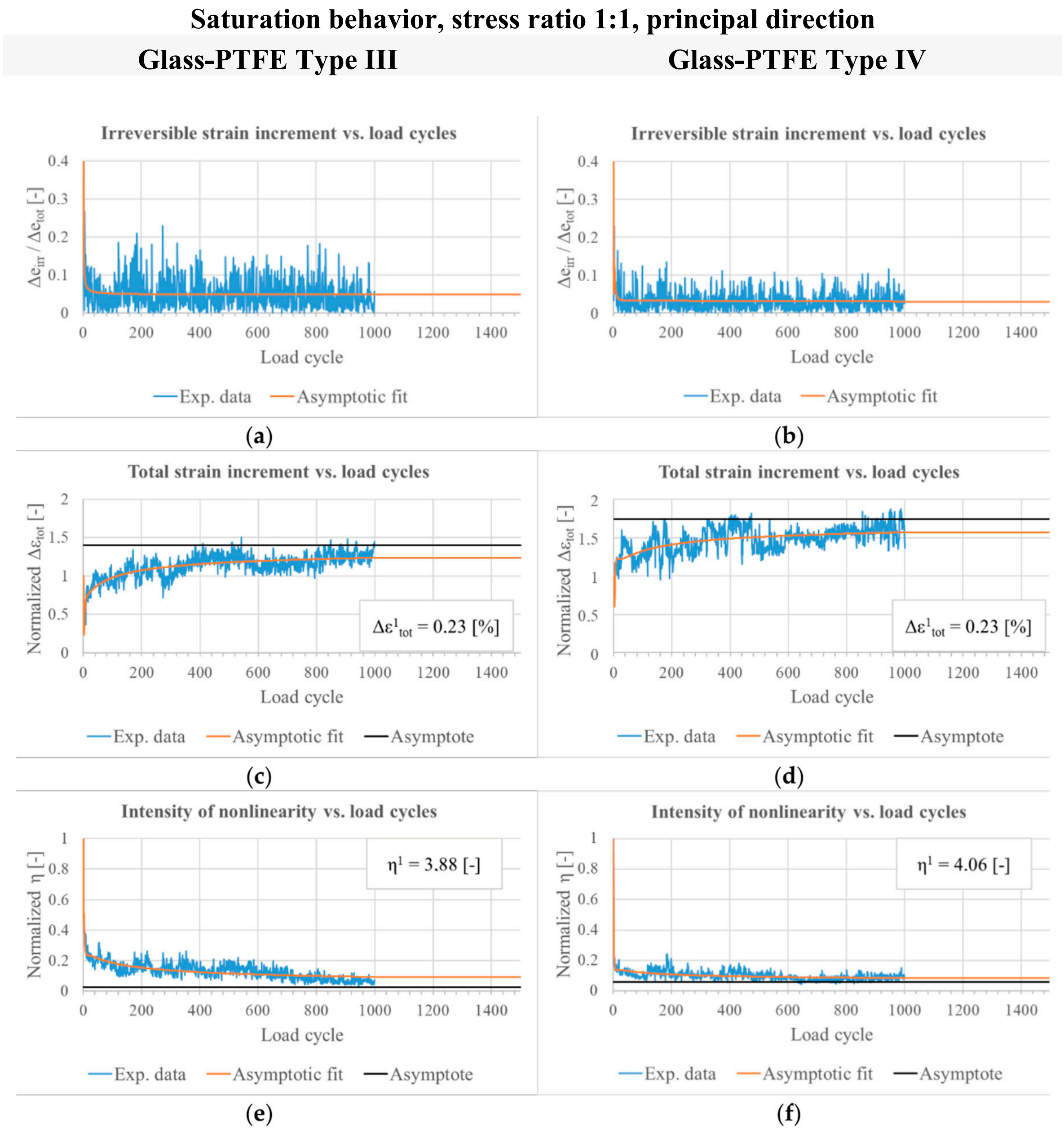

Figure S1. Curves of the saturation inspection characteristics for stress ratio 1:1, principal direction. (**a**) Irreversible strain increment vs. load cycles; (**b**) irreversible strain increment vs. load cycles; (**c**) total strain vs. load cycles; (**d**) total strain vs. load cycles; (**e**) intensity of nonlinearity vs. load cycles and (**f**) intensity of nonlinearity vs. load cycles.

Saturation behavior, stress ratio 1:1, transverse direction

Glass-PTFE Type III	Glass-PTFE Type IV

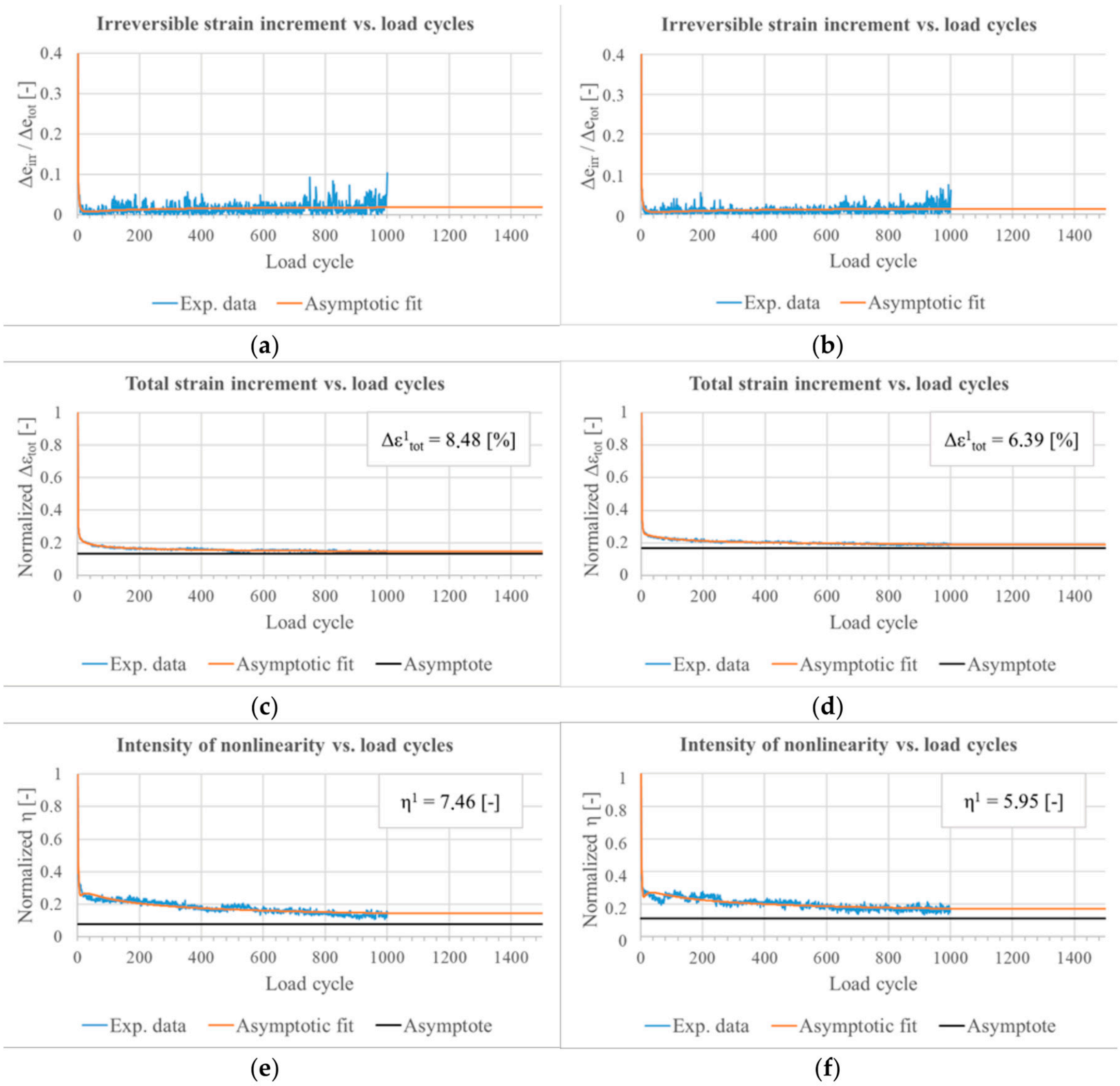

Figure S2. Curves of the saturation inspection characteristics for stress ratio 1:1, transverse direction. (**a**) Irreversible strain increment vs. load cycles; (**b**) irreversible strain increment vs. load cycles; (**c**) total strain vs. load cycles; (**d**) total strain vs. load cycles; (**e**) intensity of nonlinearity vs. load cycles and (**f**) intensity of nonlinearity vs. load cycles.

Saturation behavior, stress ratio 2:1, principal direction

Glass-PTFE Type III	Glass-PTFE Type IV

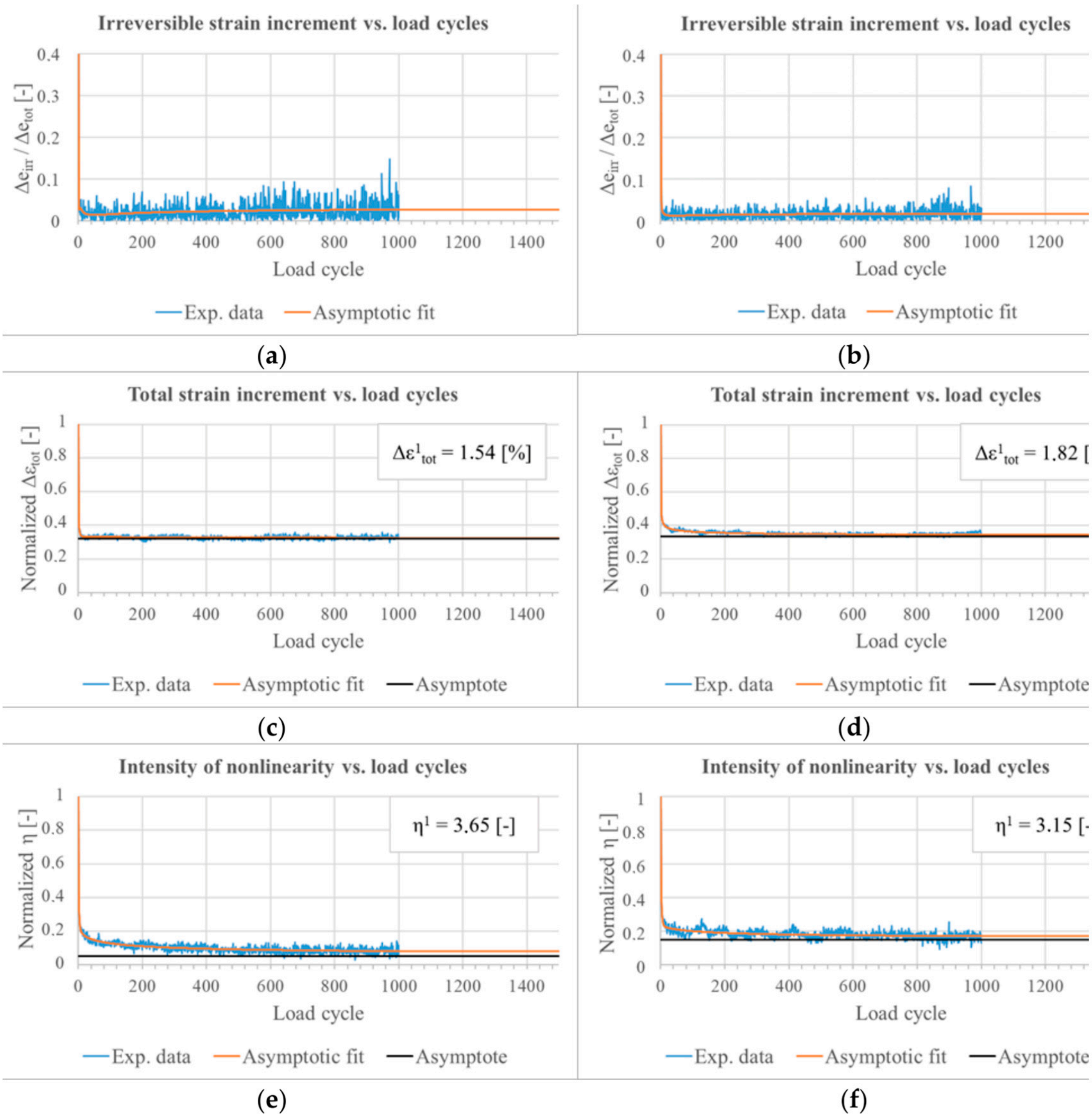

Figure S3. Curves of the saturation inspection characteristics for stress ratio 2:1, principal direction. (**a**) Irreversible strain increment vs. load cycles; (**b**) irreversible strain increment vs. load cycles; (**c**) total strain vs. load cycles; (**d**) total strain vs. load cycles; (**e**) intensity of nonlinearity vs. load cycles and (**f**) intensity of nonlinearity vs. load cycles.

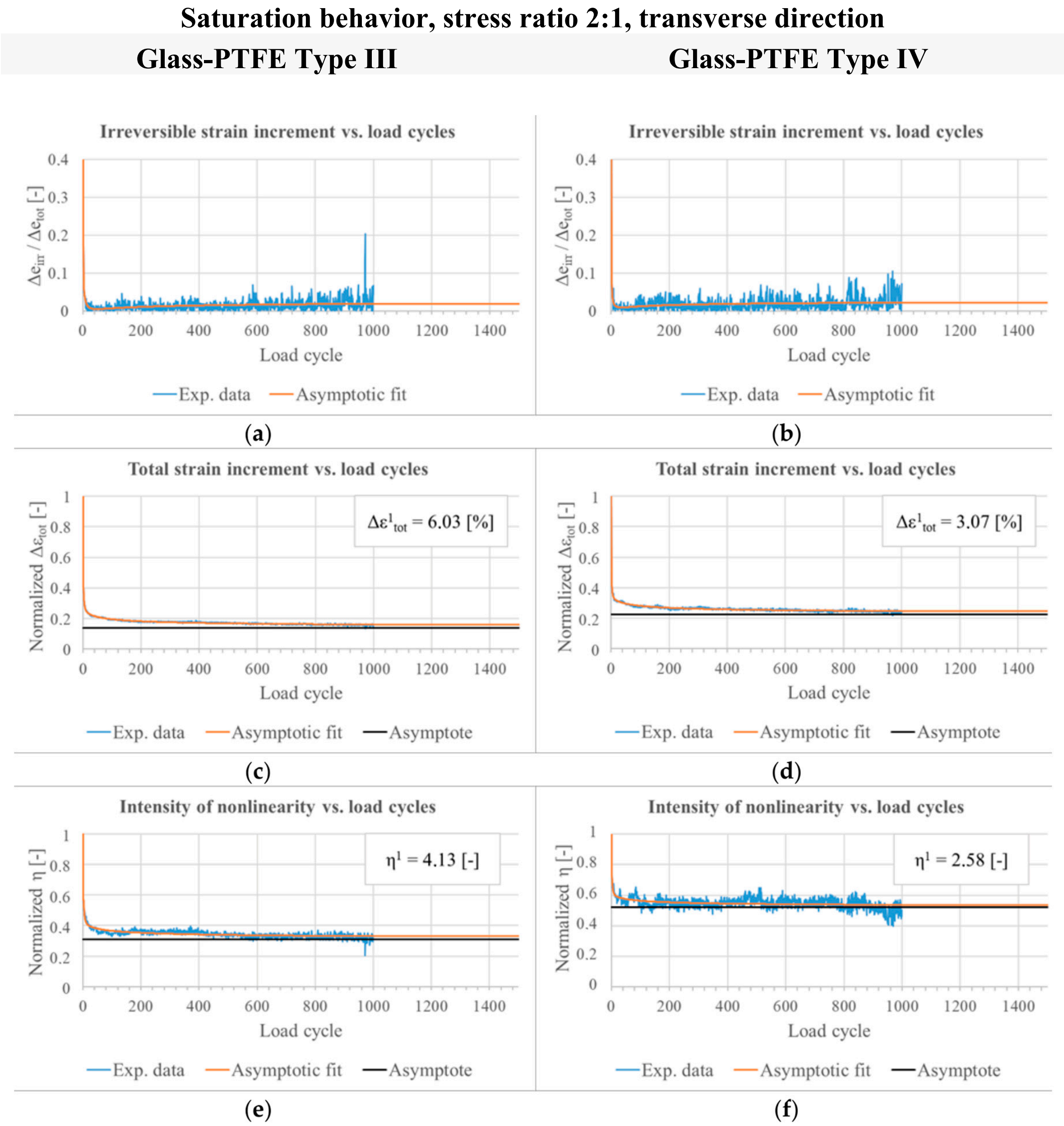

Figure S4. Curves of the saturation inspection characteristics for stress ratio 2:1, transverse direction. (**a**) Irreversible strain increment vs. load cycles; (**b**) irreversible strain increment vs. load cycles; (**c**) total strain vs. load cycles; (**d**) total strain vs. load cycles; (**e**) intensity of nonlinearity vs. load cycles and (**f**) intensity of nonlinearity vs. load cycles.

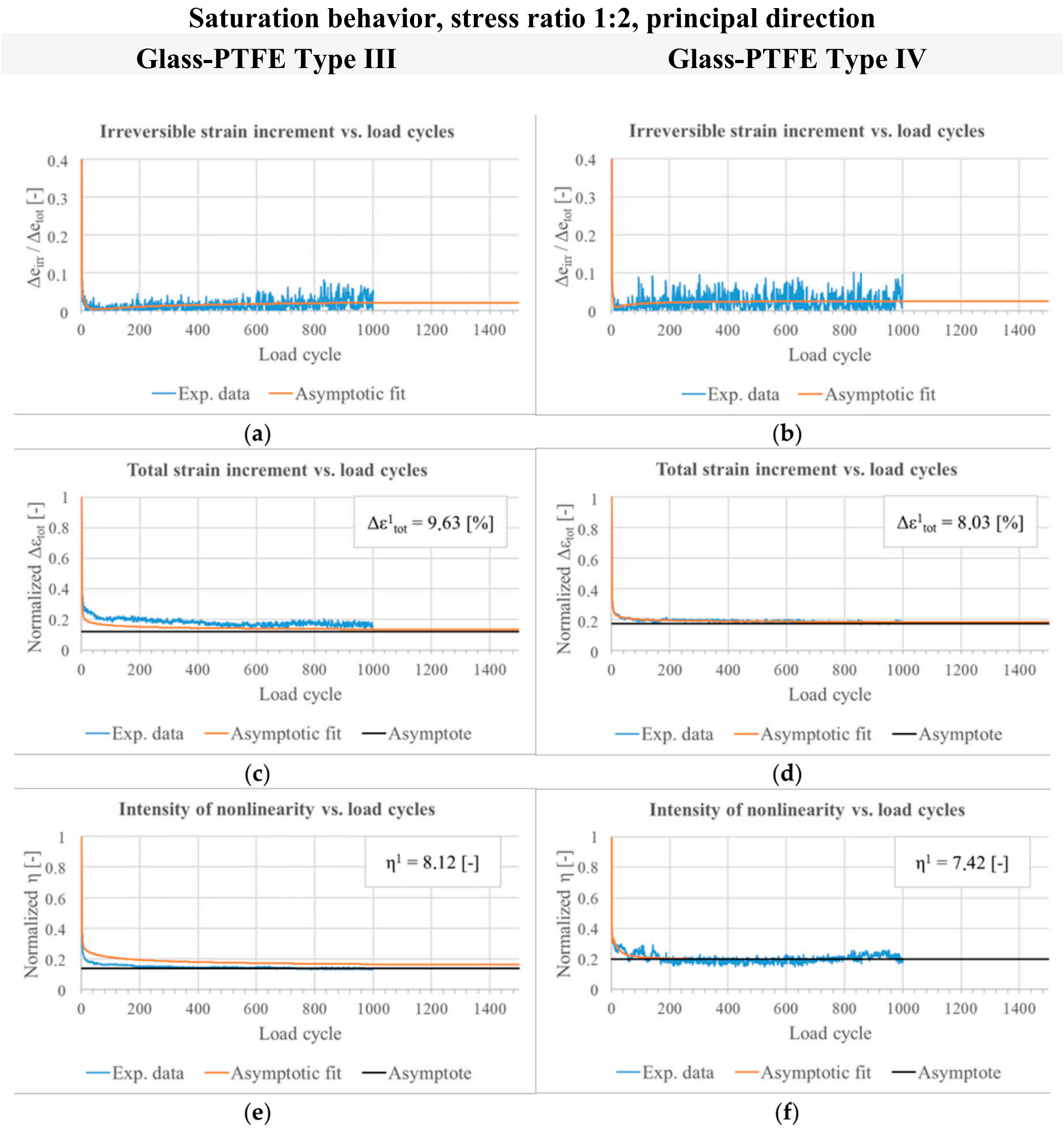

Figure S5. Curves of the saturation inspection characteristics for stress ratio 1:2, principal direction. (**a**) Irreversible strain increment vs. load cycles; (**b**) irreversible strain increment vs. load cycles; (**c**) total strain vs. load cycles; (**d**) total strain vs. load cycles; (**e**) intensity of nonlinearity vs. load cycles and (**f**) intensity of nonlinearity vs. load cycles.

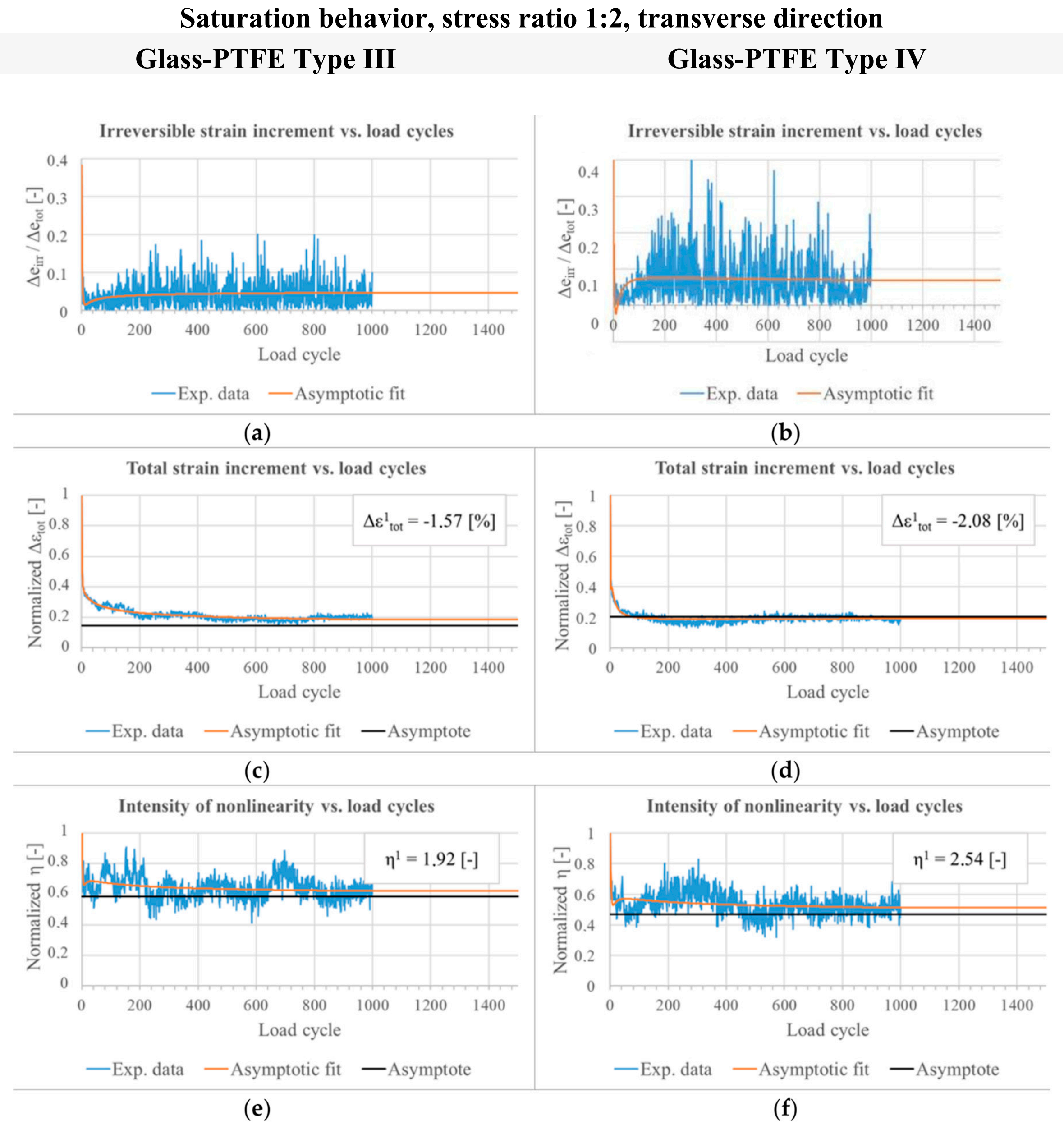

Figure S6. Curves of the saturation inspection characteristics for stress ratio 1:2, transverse direction. (**a**) Irreversible strain increment vs. load cycles; (**b**) irreversible strain increment vs. load cycles; (**c**) total strain vs. load cycles; (**d**) total strain vs. load cycles; (**e**) intensity of nonlinearity vs. load cycles and (**f**) intensity of nonlinearity vs. load cycles.

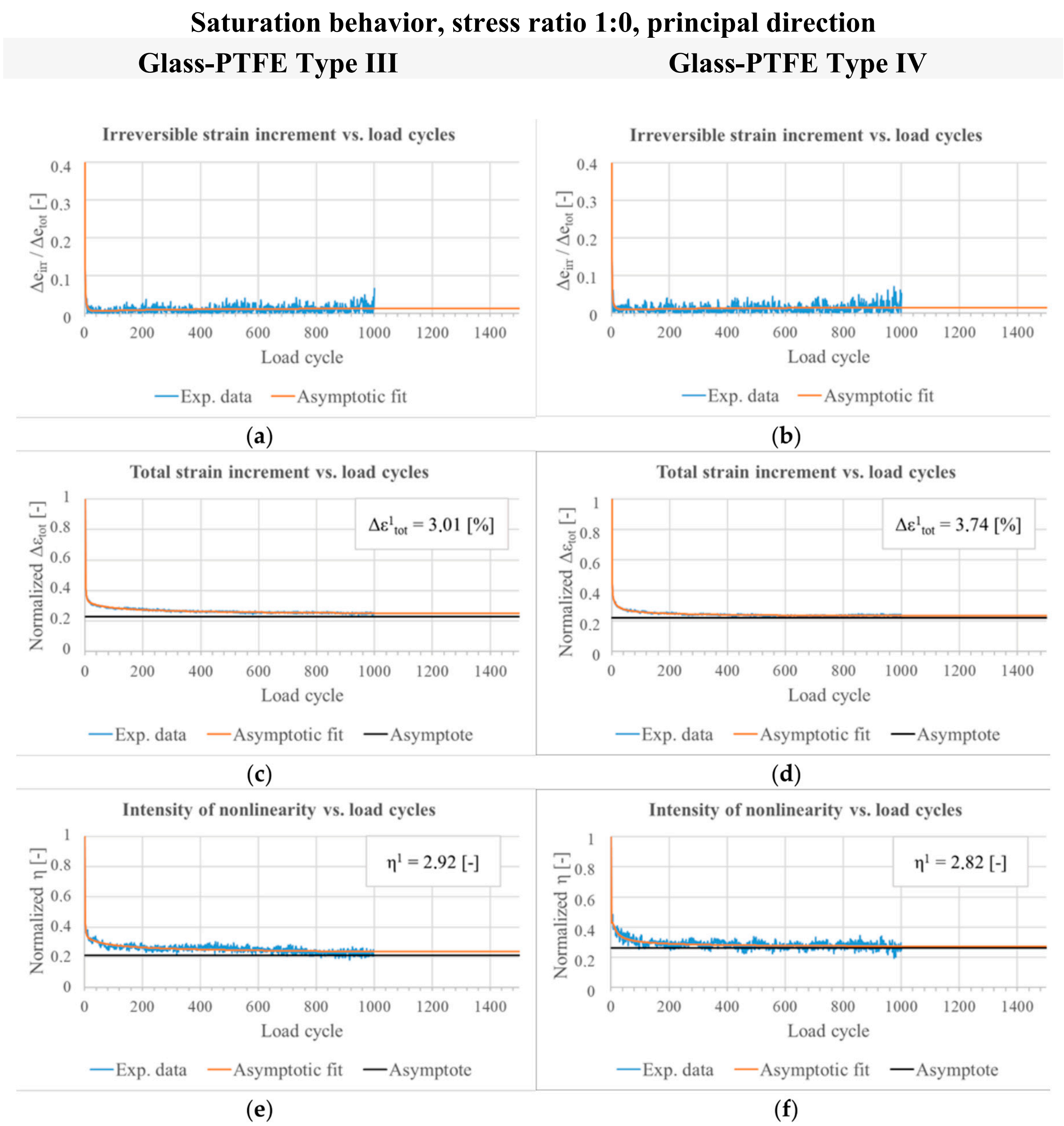

Figure S7. Curves of the saturation inspection characteristics for stress ratio 1:0, principal direction. (**a**) Irreversible strain increment vs. load cycles; (**b**) irreversible strain increment vs. load cycles; (**c**) total strain vs. load cycles; (**d**) total strain vs. load cycles; (**e**) intensity of nonlinearity vs. load cycles and (**f**) intensity of nonlinearity vs. load cycles.

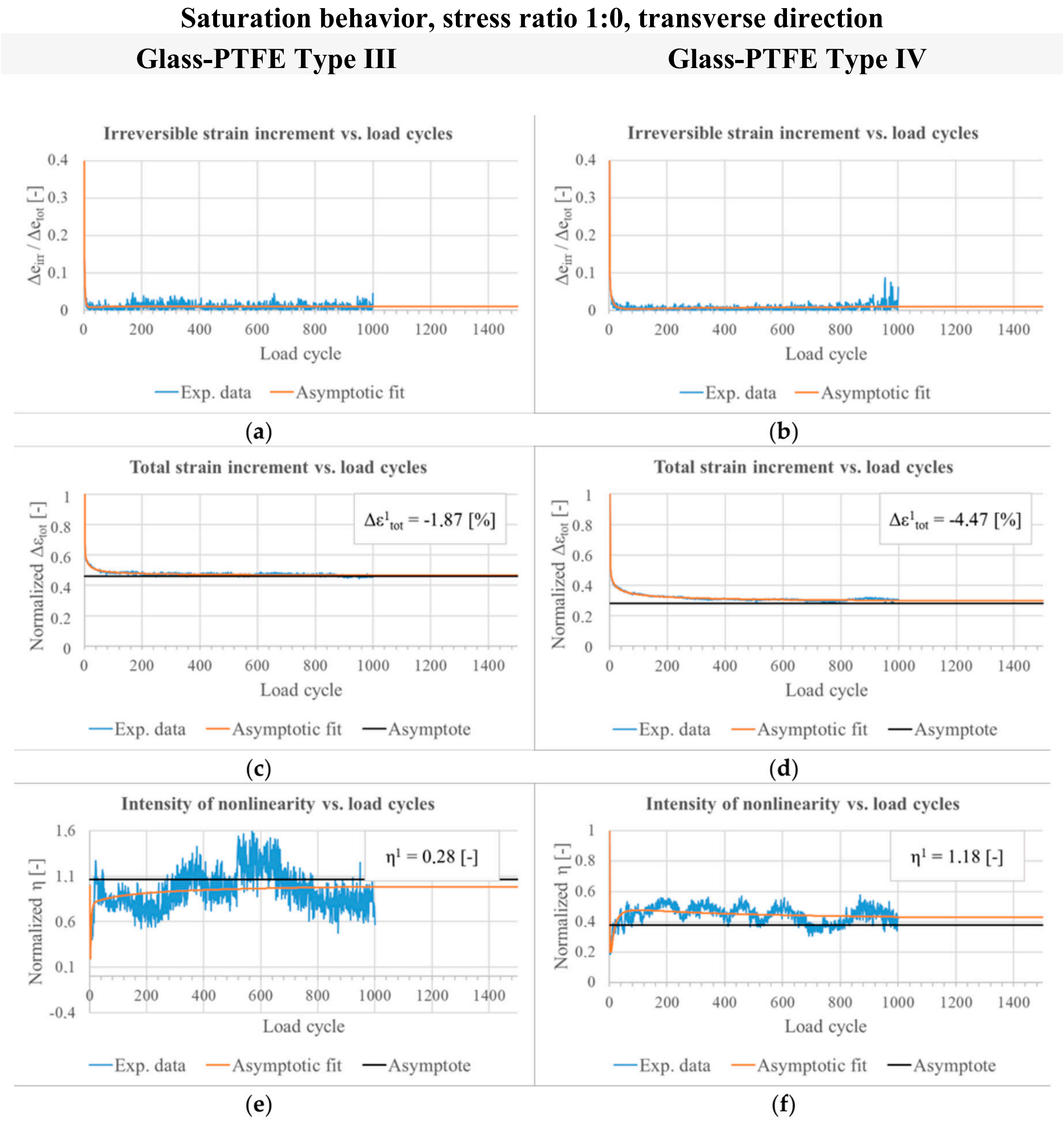

Figure S8. Curves of the saturation inspection characteristics for stress ratio 1:0, transverse direction. (**a**) Irreversible strain increment vs. load cycles; (**b**) irreversible strain increment vs. load cycles; (**c**) total strain vs. load cycles; (**d**) total strain vs. load cycles; (**e**) intensity of nonlinearity vs. load cycles and (**f**) intensity of nonlinearity vs. load cycles.

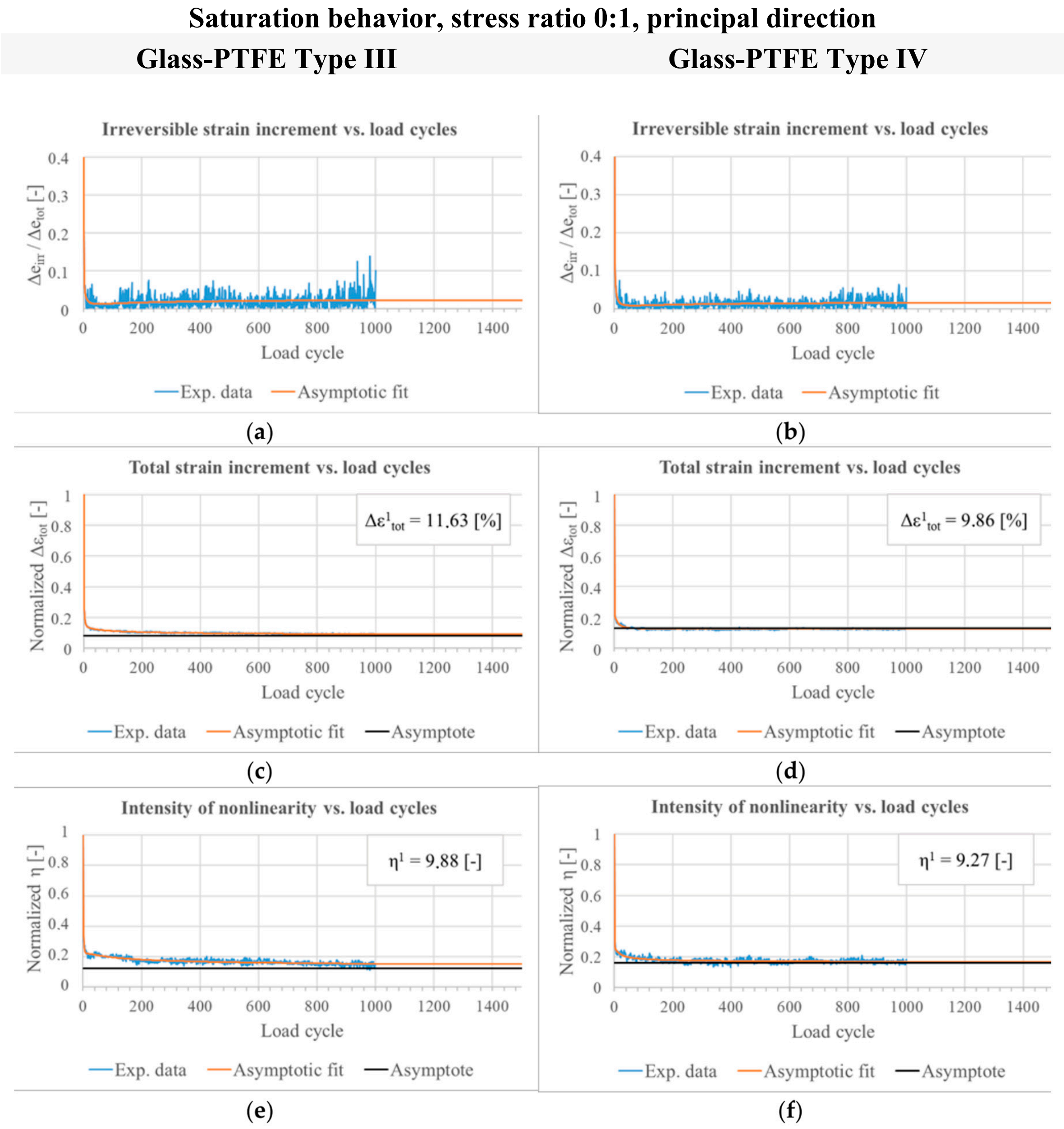

Figure S9. Curves of the saturation inspection characteristics for stress ratio 0:1, principal direction. (**a**) Irreversible strain increment vs. load cycles; (**b**) irreversible strain increment vs. load cycles; (**c**) total strain vs. load cycles; (**d**) total strain vs. load cycles; (**e**) intensity of nonlinearity vs. load cycles and (**f**) intensity of nonlinearity vs. load cycles.

Saturation behavior, stress ratio 0:1, transverse direction

Glass-PTFE Type III | **Glass-PTFE Type IV**

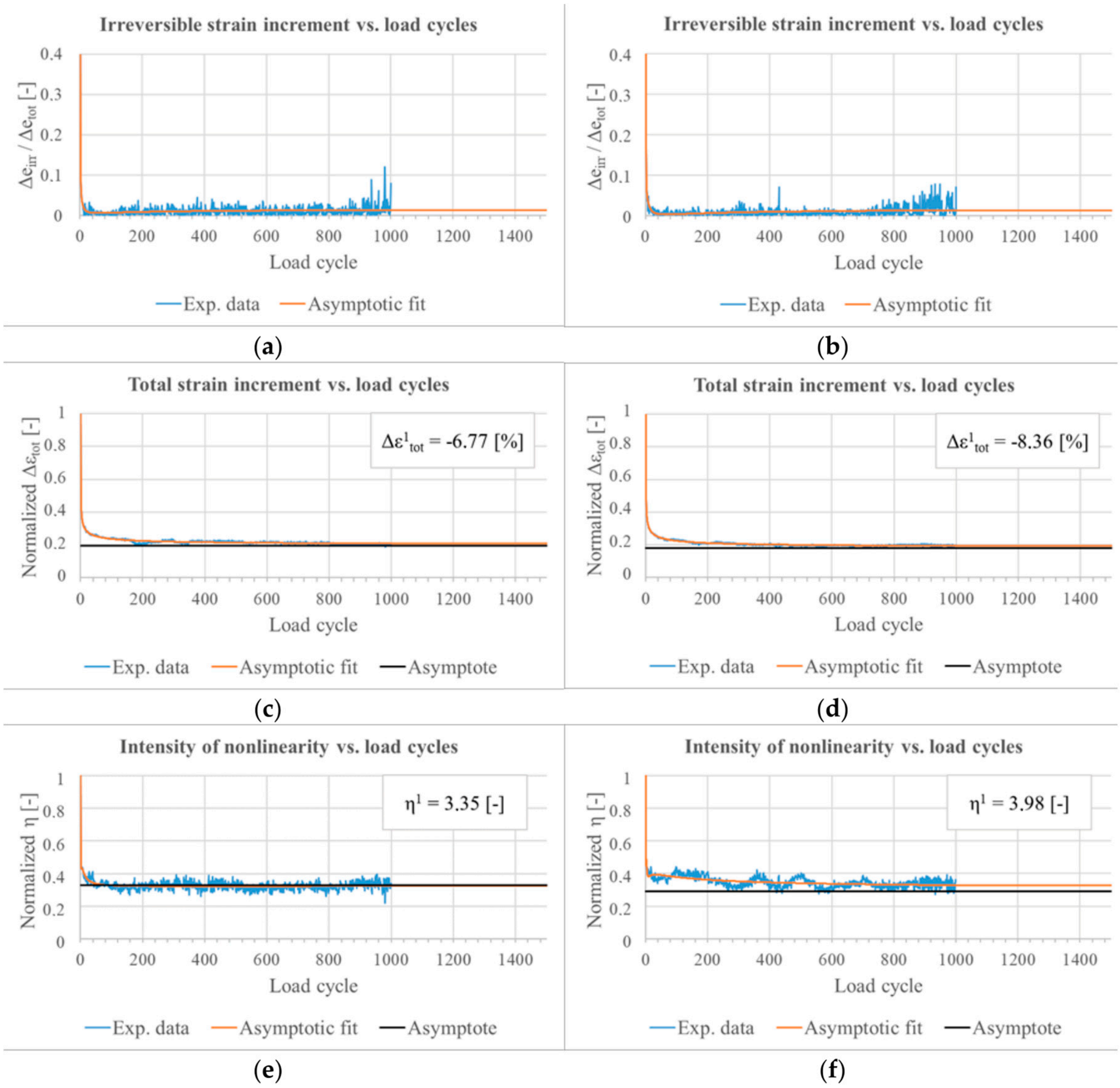

Figure S10. Curves of the saturation inspection characteristics for stress ratio 0:1, transverse direction. (**a**) Irreversible strain increment vs. load cycles; (**b**) irreversible strain increment vs. load cycles; (**c**) total strain vs. load cycles; (**d**) total strain vs. load cycles; (**e**) intensity of nonlinearity vs. load cycles and (**f**) intensity of nonlinearity vs. load cycles.
